# Point-of-care artificial intelligence-enabled ECG for dyskalemia: a retrospective cohort analysis for accuracy and outcome prediction

**DOI:** 10.1038/s41746-021-00550-0

**Published:** 2022-01-19

**Authors:** Chin Lin, Tom Chau, Chin-Sheng Lin, Hung-Sheng Shang, Wen-Hui Fang, Ding-Jie Lee, Chia-Cheng Lee, Shi-Hung Tsai, Chih-Hung Wang, Shih-Hua Lin

**Affiliations:** 1 Medical Technology Education Center, School of Medicine, National Defense Medical Center, Taipei, Taiwan, ROC; 2 Graduate Institute of Life Sciences, National Defense Medical Center, Taipei, Taiwan, ROC; 3 School of Public Health, National Defense Medical Center, Taipei, Taiwan, ROC; 4 Department of Medicine, Providence St. Vincent Medical Center, Portland, OR USA; 5 Division of Cardiology, Department of Internal Medicine, Tri-Service General Hospital, National Defense Medical Center, Taipei, Taiwan, ROC; 6 Division of Clinical Pathology, Department of Pathology, Tri-Service General Hospital, National Defense Medical Center, Taipei, Taiwan, ROC; 7 Department of Family and Community Medicine, Department of Internal Medicine, Tri-Service General Hospital, National Defense Medical Center, Taipei, Taiwan, ROC; 8 Division of Nephrology, Department of Medicine, Tri-Service General Hospital, National Defense Medical Center, Taipei, Taiwan, ROC; 9 Department of Medical Infromatics, Tri-Service General Hospital, National Defense Medical Center, Taipei, Taiwan, ROC; 10 Division of Colorectal Surgery, Department of Surgery, Tri-Service General Hospital, National Defense Medical Center, Taipei, Taiwan, ROC; 11 Department of Emergency Medicine, Tri-Service General Hospital, National Defense Medical Center, Taipei, Taiwan, ROC; 12 Department of Otolaryngology-Head and Neck Surgery, Tri-Service General Hospital, National Defense Medical Center, Taipei, Taiwan, ROC; 13 Graduate Institute of Medical Sciences, National Defense Medical Center, Taipei, Taiwan, ROC

**Keywords:** Evoked potentials, Diagnostic markers

## Abstract

Dyskalemias are common electrolyte disorders associated with high cardiovascular risk. Artificial intelligence (AI)-assisted electrocardiography (ECG) has been evaluated as an early-detection approach for dyskalemia. The aims of this study were to determine the clinical accuracy of AI-assisted ECG for dyskalemia and prognostic ability on clinical outcomes such as all-cause mortality, hospitalizations, and ED revisits. This retrospective cohort study was done at two hospitals within a health system from May 2019 to December 2020. In total, 26,499 patients with 34,803 emergency department (ED) visits to an academic medical center and 6492 ED visits from 4747 patients to a community hospital who had a 12-lead ECG to estimate ECG-K^+^ and serum laboratory potassium measurement (Lab-K^+^) within 1 h were included. ECG-K^+^ had mean absolute errors (MAEs) of ≤0.365 mmol/L. Area under receiver operating characteristic curves for ECG-K^+^ to predict moderate-to-severe hypokalemia (Lab-K^+^ ≤3 mmol/L) and moderate-to-severe hyperkalemia (Lab-K^+^ ≥ 6 mmol/L) were >0.85 and >0.95, respectively. The U-shaped relationships between K^+^ concentration and adverse outcomes were more prominent for ECG-K^+^ than for Lab-K^+^. ECG-K^+^ and Lab-K^+^ hyperkalemia were associated with high HRs for 30-day all-cause mortality. Compared to hypokalemic Lab-K^+^, patients with hypokalemic ECG-K^+^ had significantly higher risk for adverse outcomes after full confounder adjustment. In addition, patients with normal Lab-K^+^ but dyskalemic ECG-K^+^ (pseudo-positive) also exhibited more co-morbidities and had worse outcomes. Point-of-care bloodless AI ECG-K^+^ not only rapidly identified potentially severe hypo- and hyperkalemia, but also may serve as a biomarker for medical complexity and an independent predictor for adverse outcomes.

## Introduction

Potassium (K^+^) is the principal intracellular cation and functions to maintain the electrical gradient across cell membranes. The concentration of K^+^ in plasma is determined by the distribution of K^+^ between intracellular and extracellular fluid (ECF) and by renal K^+^ excretion. Thus, dyskalemias, including hypokalemia and hyperkalemia, usually arises from derangements of transcellular K^+^ shift^1^ and/or defective renal/extrarenal K^+^ excretion^2^. Dyskalemia causes cardiovascular, neuromuscular, renal and metabolic disturbances, and is a common electrolyte abnormality associated with cardiovascular complications and increased mortality^3,4^. Up to 20–50% of patients hospitalized for acute illnesses have hypokalemia (plasma K^+^ value ≤3.5 mmol/L) and 2–5% have hyperkalemia (K^+^ ≥5.5 mmol/L)^5^. In our recent study, 22% and 2% of all patients visiting the emergency department (ED) had hypokalemia and hyperkalemia, respectively^6^. Traditionally, the diagnosis of dyskalemia relies on blood tests with varying turnaround times^7^.

Electrocardiography (ECG), as a prompt, non-invasive, and convenient bedside tool, may detect electrical changes associated with dyskalemias, including altered PR interval, QRS, ST-segment, T-wave amplitude, and/or corrected QT interval^8^. However, these morphologic ECG changes associated with dyskalemias may not be easily recognized even by experienced physicians or cardiologists. With the revolution and advancement of artificial intelligence (AI) techniques, deep learning models (DLM)^9^ have been shown to achieve human-level performance and effectively detect cardiac diseases with large annotated ECG datasets^10–16^. Based on analysis of an annotated 66,000-ECG dataset, we have successfully used DLM to develop ECG12Net for the rapid quantitative estimation of blood K^+^ concentration to detect dyskalemias in the ED^6^. However, a “black-box pragmatic study“^17^ to validate the accuracy of bloodless detection of dyskalemias based on AI-ECG is still warranted before integration into routine clinical workflows.

Laboratory dyskalemia is widely known to be associated with higher morbidity and mortality in populations with different diseases such as diabetes mellitus, chronic kidney disease, acute myocardial infarction and chronic heart failure^3,5,18–21^. However, the clinical applicability and significance of AI-enhanced ECG for the detection of dyskalemia remains unclear. Moreover, the association between ECG-dyskalemia and outcomes has not been evaluated. Since ECG is more germane to the underlying pathophysiological and electrical changes in the heart, both systemic and cardiac disorders may cause abnormal ECGs. We hypothesize that abnormal ECG-K^+^ with normal Lab-K^+^ may be a surrogate for underlying cardiac conditions portend worse outcomes than patients with both normal values.

In this retrospective cohort over 1.5 years, we aimed to determine the clinical accuracy of real-time AI-assisted ECG for dyskalemia with external validation and examined patient outcomes in a large cohort with different combinations of ECG-K^+^ and Lab-K^+^. As shown in the Graphical abstract, our AI-enabled ECG system rapidly calculated ECG-K^+^ for point-of-care support in the ED and achieved comparable accuracy to previous studies^6,10,22^ in detecting moderate-to severe dyskalemia. In addition, when ECG-K^+^ and Lab-K^+^ were discordant, ECG-K^+^ was more predictive of subsequent all-cause mortality, hospitalizations, and ED revisits.

## Results

### Timing and incidence of ECG and lab-based dyskalemia

**Fig. 1:**
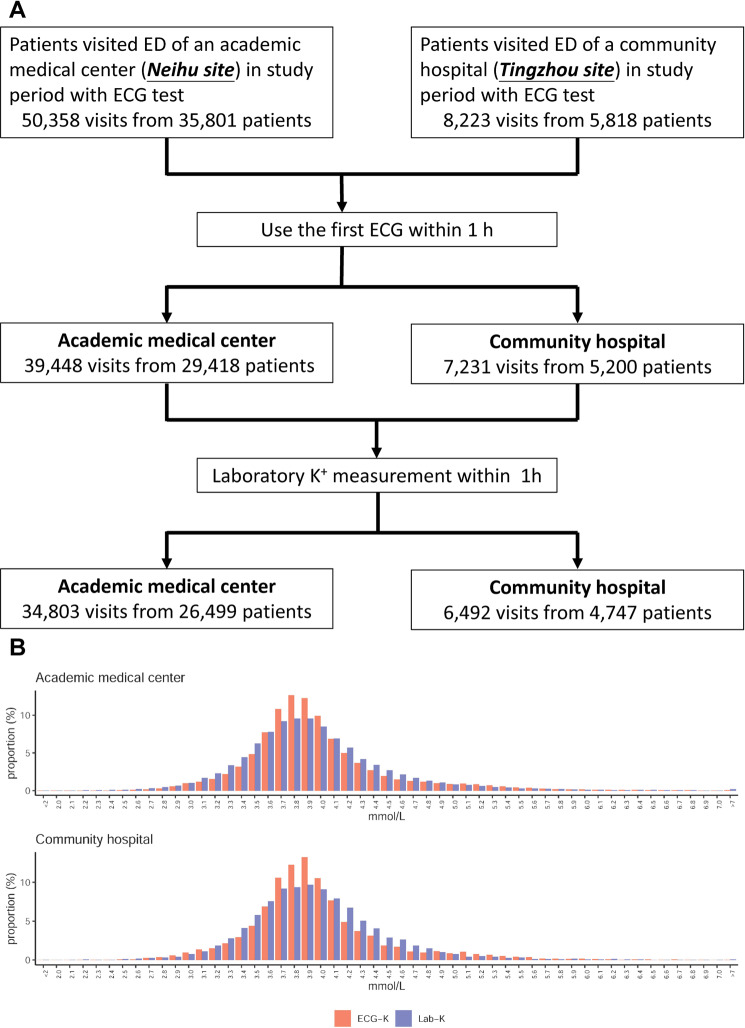
Study cohorts’ summary. **A** Dataset generation based on emergency department visits to an academic medical center and a community hospital; (**B**) The distributions of ECG-K^+^ and Lab-K^+^ at the academic medical center and community hospital.

At the medical center, the wait time between order entry and ECG test and blood draw were 21.5 ± 17.9 and 26.2 ± 9.3 min, respectively; the Lab-K^+^ resulted at 64.3 ± 16.5 min. At the community hospital, the wait time for ECG and blood draw were 20.8 ± 15.2 and 24.6 ± 9.1 min, respectively, and the Lab-K^+^ resulted at 54.9 ± 15.5 min. The ECG-K^+^ was available much sooner than the Lab-K^+^ at both the academic medical center (42.8 ± 22.5 min) and community hospital (34.1 ± 18.8 min). For Lab-K^+^, there were 7,313 (21.0%) visits with mild to severe hypokalemia and 745 (2.1%) visits with mild to severe hyperkalemia at the academic medical center. There were 1151 (17.7%) visits with mild to severe hypokalemia and 92 (1.4%) visits with mild to severe hyperkalemia at the community hospital. For ECG-K^+^, there were 4331/719 (12.4%/2.1%) visits with ECG-K^+^ ≤3.5 mmol/L/ECG-K^+^ ≥ 5.5 mmol/L at the academic medical center and 778/114 (12.0%/1.8%) visits at the community hospital. Compared to Lab-K^+^, the distribution of ECG-K^+^ was more concentrated (Fig. 1B), suggesting that abnormal values of ECG-K^+^ might be more important due to the algorithm’s tendency to make more conservative predictions.

### Accuracy of AI-ECG detection for dyskalemia

**Fig. 2:**
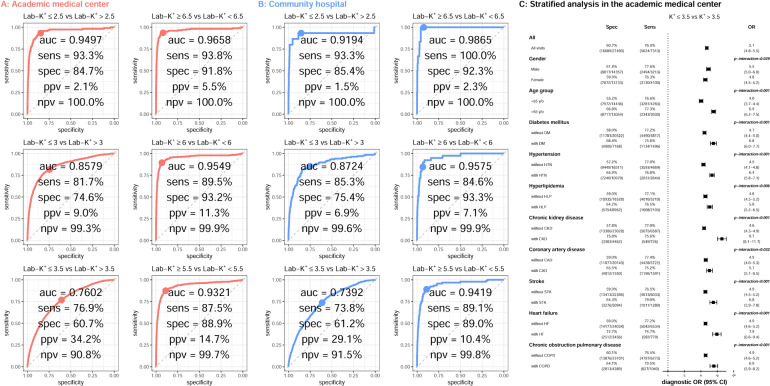
Performance of ECG-K^+^ for detecting mild to severe hypo/hyperkalemia. The ROC curves for varying degrees of hypo- and hyperkalemia at the academic medical center (**A**) and community hospital (**B**). The cut-of-points for each plot were defined previously, which are the same between the two hospitals. Stratified analyses with the most significant differences are shown in (**C**).

The mean absolute errors (MAEs) of Lab-K^+^ and ECG-K^+^ were 0.365 and 0.364 mmol/L at the medical center and community hospital, respectively. The ECG-K^+^ calculated with 12 leads was more accurate than using any one lead (Supplementary Fig. 1). As shown in Fig. 2, for the detection of severe hypokalemia (Lab-K^+^ ≤2.5) and hyperkalemia (Lab-K^+^ ≥6.5), ECG-K^+^ achieved area under curves (AUCs) of 0.9497/0.9658 with sensitivities of 93.3%/93.8% and specificities of 84.7%/91.8% at the academic medical center; the AUCs were 0.9194/0.9865 with sensitivities of 93.3%/100.0% and specificities of 85.4%/92.3% at the community hospital. Although the AUCs declined modestly for the detection of moderate (Lab-K^+^ ≤3.0 or Lab-K^+^ ≥6.0) to mild (Lab-K^+^ ≤3.5 or Lab-K^+^ ≥5.5) dyskalemia, the values were still higher than 0.85 except for mild hypokalemia. The positive and negative predictive values were 29.1–34.2% and 90.8–91.5%, respectively, for detecting hypokalemia (Lab-K^+^ ≤3.5 mmol/L). Due to the low prevalence of hyperkalemia (1.4–2.1%), the positive and negative predictive values were 10.4–14.7% and 99.7–99.8%, respectively, for detecting hyperkalemia (Lab-K^+^ ≥5.5 mmol/L). As shown in Supplementary Fig. 2, mild hypokalemia as predicted by ECG-K^+^ was significantly associated with increasing age and co-morbidities including diabetes mellitus (DM), hypertension (HTN), chronic kidney disease (CKD), stroke (STK), heart failure (HF), and chronic obstruction pulmonary disease (COPD). This suggests that these patients are likely sicker with underlying organ dysfunction than patients with lab-hypokalemia but normal ECG-K^+^ (false negative). ECG-K^+^ performance was strong across all degrees of hyperkalemia. Comparing the performance of 12 leads versus each lead, the AI-enabled algorithm using 12 leads was more accurate in all analyses (Supplementary Fig. 3).

### Patients’ characteristics in lab and AI-ECG dyskalemia

Patients’ characteristics are shown in Supplementary Table 1. Between the academic medical center and community hospital, the prevalence of co-morbidities were HTN 39.1%/51.4%, hyperlipidemia (HLP) 31.8%/43.1%, coronary artery disease (CAD) 25.8%/32.0%, DM 24.9%/32.5%, STK 18.3%25.0%, COPD 15.6%/27.6%, CKD 14.9%/15.2%, and HF 12.2%/14.9%. Elevated blood sugar, creatinine, CRP, PCT, pBNP, D-dimer, and proteinuria were frequently observed in these ED visits (40.1%/42.8%), suggesting that each ED visit was related to different stress conditions, such as acute or chronic renal failure, infections with or without sepsis, cardiovascular, cardiopulmonary, or metabolic complications.

**Fig. 3:**
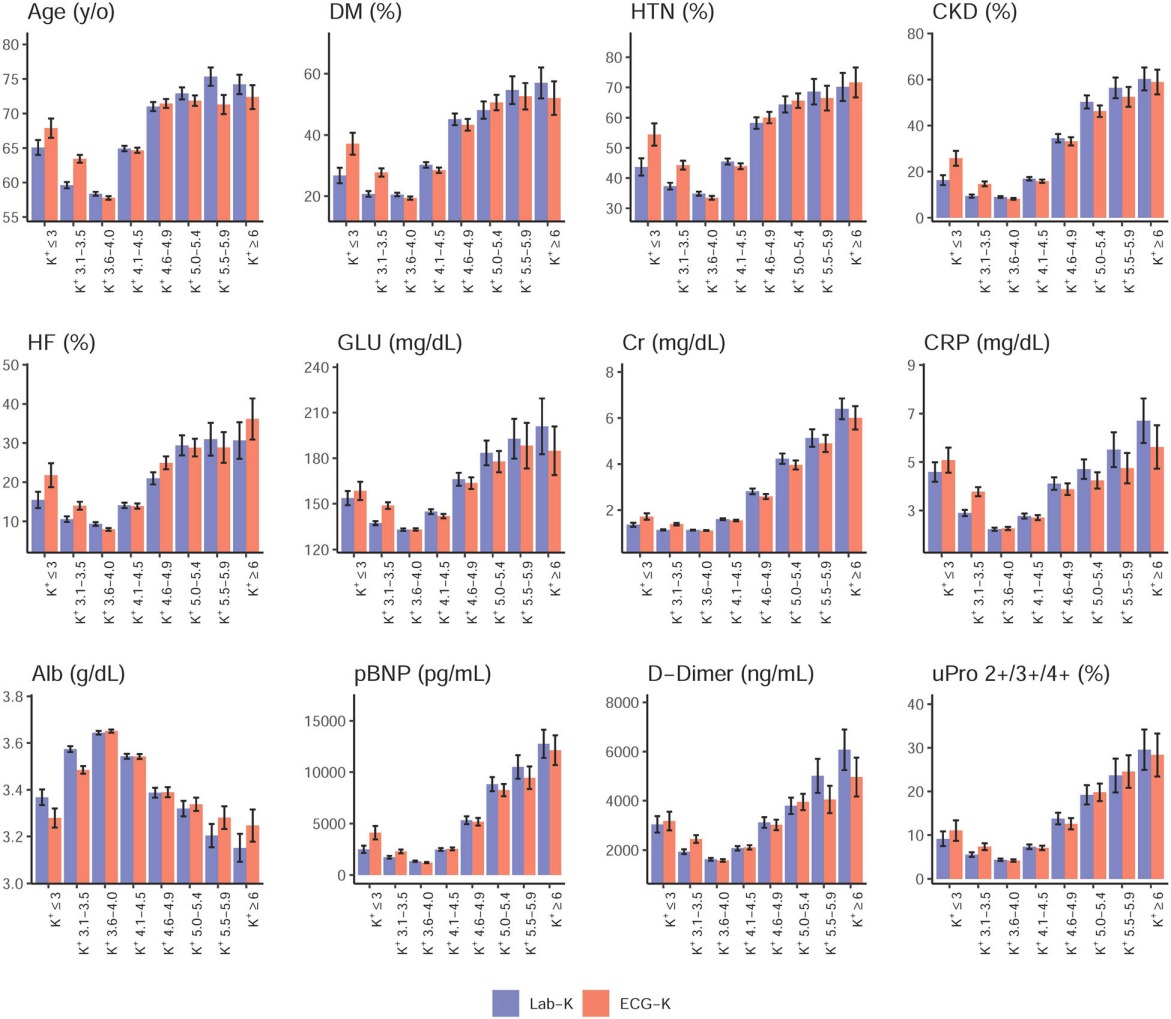
Distributions of selected patients’ characteristics in each ECG-K^+^ and Lab-K^+^ group. Bars represent the mean or proportion where appropriate and corresponding 95% conference intervals, which are adjusted by hospital.

Patients with hyperkalemic Lab-K^+^ (≥5.5 mmol/L) exhibited significantly greater age, predilection for DM, HTN, CKD, CAD, HF, and lower blood pressure than patients with normal Lab-K^+^ (3.6–5.4 mmol/L) and hypokalemic Lab-K^+^ (≤3.5 mmol/L) (Fig. 3). Significantly higher plasma creatinine, blood urea nitrogen (BUN), NT-pro-B type natriuretic peptide (pBNP), D-dimer, glucose, procalcitonin (PCT), C-reactive protein (CRP), SGOT/SGPT and lower hemoglobin, albumin, sodium (Na^+^), bicarbonate (HCO_3_) and blood pH value were also observed. Patients with hypokalemic Lab-K^+^ did not show significant differences compared to those with normal Lab-K^+^ with regards to age, co-morbidities (DM, HTN, HLP, CKD, CHF, and CAD), and most laboratory data (renal function, liver function, pBNP, glucose, D-dimer, hemoglobin, albumin, HCO_3_, and blood pH value) except for slightly higher white blood count (WBC) and CRP.

Like hyperkalemic Lab-K^+^, patients with predicted hyperkalemic ECG-K^+^ (≥5.5 mmol/L) were more complex, including older age, more co-morbidities (DM, HTN, CKD, and HF) and significantly higher glucose, CRP, pBNP, D-dimer, proteinuria, and lower serum albumin compared with those with normal ECG-K^+^ (3.6–5.4 mmol/L) or hypokalemia (≤3.5 mmol/L) (Fig. 3). Patients with hypokalemic ECG-K^+^ had significantly higher glucose, CRP, pBNP, D-dimer, proteinuria, and lower serum albumin compared to those with normal ECG-K^+^. Compared to hypokalemic Lab-K^+^, patients with hypokalemic ECG-K^+^ had older age, more co-morbidities (DM, HTN, CKD, and HF) with significantly higher glucose, CRP, pBNP, D-dimer, proteinuria and lower serum albumin. Details of the relationship between other patients’ characteristics and K^+^ concentrations are shown in Supplementary Fig. 4.

### Risk relationship between AI-ECG and lab dyskalemia

**Fig. 4:**
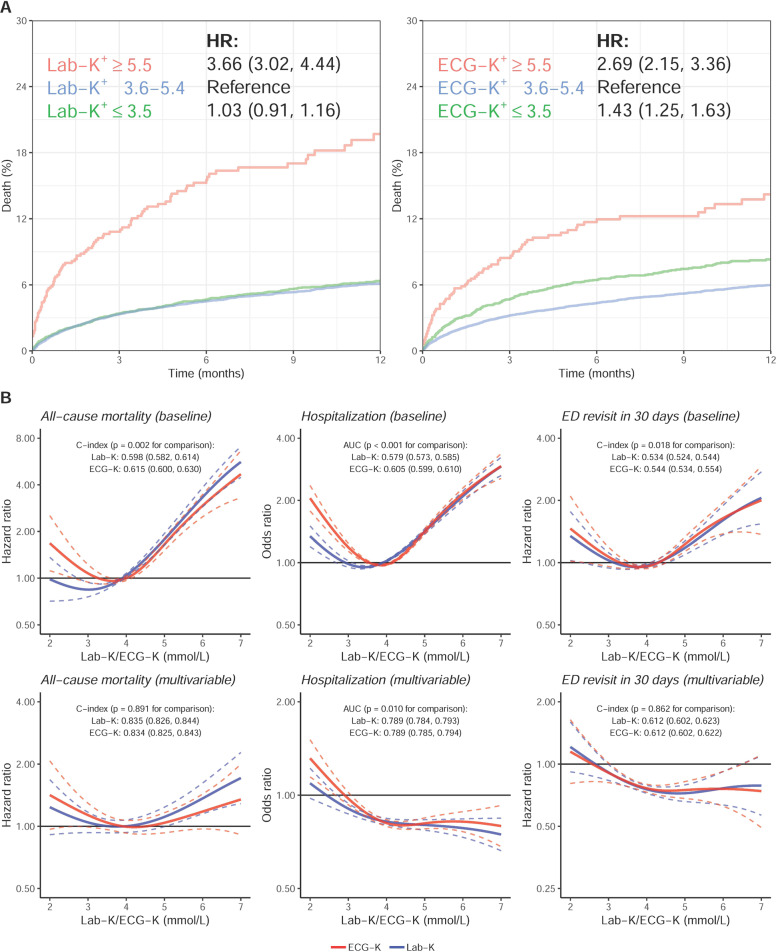
The relationship between ECG-K^+^ and Lab-K^+^ on adverse outcomes in combined analysis from both hospitals. **A** The Kaplan–Meier curve analysis of all-cause mortality in hypo- and hyperkalemia as defined by ECG-K^+^ and Lab-K^+^. The hazard ratio (HR) was adjusted by hospital site; (**B**) Continuous association of ECG-K^+^ and Lab-K^+^ on adverse outcomes. The solid line and dashed line are point estimation and corresponding 95% conference interval, respectively. The baseline model of combined analysis is adjusted to each hospital site and based on Cox proportional hazard model or logistic regression as appropriate for each outcome. The multivariable analyses include significant variables in risk-effect analyses (All-cause mortality: gender, Age, SBP, DBP, HLP, Hb, HCO_3_, Blood pH, Na, AST, ALT, Alb, CRP, pBNP, and D-dimer; Hospitalization: gender, age, BMI, DBP, smoke, HLP, STK, HF, WBC, Hb, PLT, HCO_3_, PH, Na, Cl, tCa, GLU, AST, CK, Alb, CRP, TnI, and D-dimer; ED revisits in 30 days: gender, DM, CAD, STK, COPD, Hb, and Na).

During a median follow-up of 3.9 months (interquartile range: 0.5–10.7 months) for the medical center and 4.5 months (0.5–11.2 months) for the community hospital, the gross mortality rates were 4.1% (1414/34,803) and 2.8% (179/6492) at the medical center and community hospital, respectively. There was a significantly increased risk of death (HR 3.66, 95% CI 3.02–4.44) in patients with hyperkalemic Lab-K^+^ (≥5.5 mmol/L) but not in those with hypokalemic Lab-K^+^ (≤3.5 mmol/L) [hazard ratio (HR) 1.03, 95% confidence interval (CI) 0.91–1.16] compared with normokalemia (Lab-K^+^ 3.6–5.4 mmol/L) (Fig. 4A). Using the ECG-K^+^, increased mortality was seen in both ECG-hypokalemia (HR 1.43, 95% CI 1.25-1.63) and ECG-hyperkalemia (HR 2.69, 95% CI 2.15–3.36). The relationships between all-cause mortality and the range of measured Lab-K^+^ and predicted ECG-K^+^ from 2 to 7 mmol/L are shown in Fig. 4B. The lowest risk for all-cause mortality occurred in Lab-K^+^ between 2 and 4.5 mmol/L, with increasing risk above 4.5 mmol/L and the highest risk for Lab-K^+^ ≥6 mmol/L (more than fourfold risk). The relationship between ECG-K^+^ and all-cause mortality revealed a prominent U-shape, with ECG-K^+^ <3 mmol/L carrying higher risk. The concordance index (C-index) of ECG-K^+^ for all-cause mortality in the baseline model was 0.615 (95% CI 0.600–0.630), which was significantly better than Lab-K^+^ (0.598, 95% CI 0.582–0.614, *p* = 0.002).

We further explored the reasons behind the predictive ability of ECG-K^+^. Supplementary Fig. 5 shows the risk-effect analyses of patient characteristics with Lab-K^+^ and ECG-K^+^. Gender, age, systolic blood pressure (SBP), diastolic blood pressure (DBP), HLP, hemoglobin (Hb), HCO_3_, blood pH, sodium (Na^+^), aspartate aminotransferase (AST), alanine aminotransferase (ALT), Alb, CRP, pBNP, and D-dimer were independently correlated with all-cause mortality. After adjusting for these risk factors, the C-index of ECG-K^+^ (C-index 0.834, 95% CI 0.826–0.843) was no longer significantly different (*p* = 0.891) from that of Lab-K^+^ (C-index 0.835, 95% CI 0.826–0.844).

The hospitalization rates were 38.3% (13,330/34,803) and 37.8% (2452/6492) at the medical center and community hospital, respectively. In the hospitalization analysis (Fig. 4B), both ECG-K^+^ and Lab-K^+^ showed a U-shaped relationship between K^+^ and the risk of hospitalization. Although the risk curves between hyperkalemic values of ECG-K^+^ and Lab-K^+^ and hospitalization were similar, the risk between hypokalemic ECG-K^+^ and hospitalization was more pronounced than that of Lab-K^+^. The AUC of ECG-K^+^ on hospitalization in the baseline model was 0.605 (95% CI 0.599-0.610), significantly higher (*p* < 0.001) than that of Lab-K^+^ (0.579, 95% CI 0.573–0.585). Age, gender, body mass index (BMI), smoke, DBP, HLP, STK, HF, WBC count, Hb, platelet (PLT), HCO_3_, Blood pH, Na^+^, chloride (Cl^−^), total calcium (tCa^++^), GLU, AST, creatine kinase (CK), Alb, CRP, troponin I (TnI), and D-dimer were significantly associated with the risk of hospitalization (Supplementary Fig. 5). After full adjustment, the predictive ability of ECG-K^+^ for hospitalization was still significantly higher than that of Lab-K^+^ (*p* = 0.010).

ED revisits within 30 days were 13.2% (2,836/21,473) and 15.8% (637/4,040) at the medical center and community hospital, respectively. There was a U-shaped relationship between plasma K^+^ and the probability of ED revisits (Fig. 4B). The C-index of ECG-K^+^ on ED revisits in the baseline model was 0.544 (95% CI 0.534-0.554), significantly higher (*p* = 0.018) than that of Lab-K^+^ (0.534, 95% CI 0.524–0.544). Gender, DM, CAD, STK, COPD, Hb, and Na^+^ were independent risk factors for ED revisits besides Lab-K^+^ and ECG-K^+^ (Supplementary Fig. 5). After full adjustment, the predictive ability of ECG-K^+^ for ED revisits was similar to that of Lab-K^+^.

### Characteristics and risk analysis in patients with discrepant Lab-K^+^ and ECG-K^+^

**Fig. 5:**
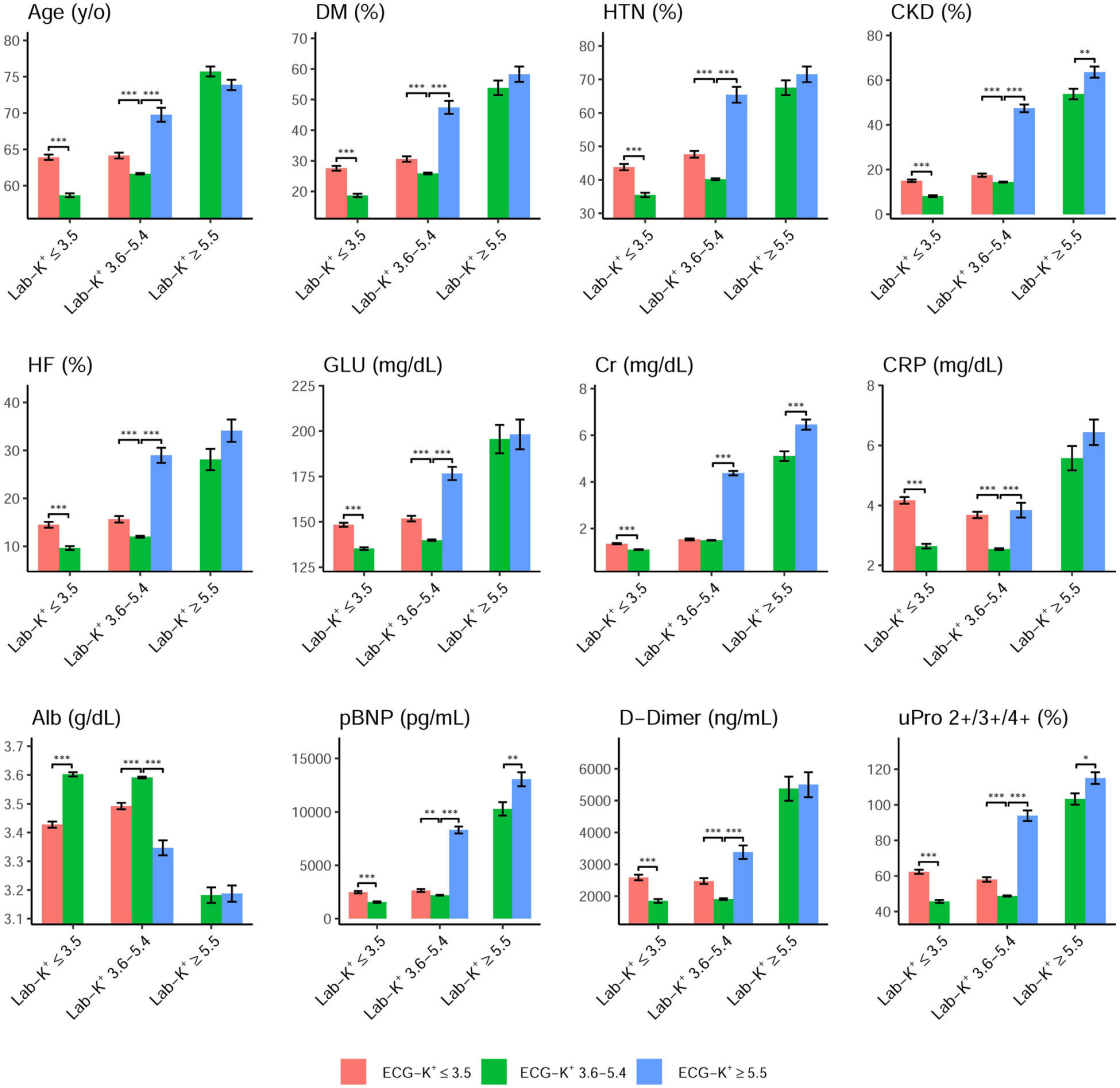
Selected patients’ characteristics in different ECG-K^+^ and Lab-K^+^ groups. Bars represent the mean or proportion where appropriate and corresponding 95% conference intervals, which are adjusted by hospital and Lab-K^+^ via linear or logistic regression (*p < 0.05; **p < 0.01; ***p < 0.001).

Patients with a normal Lab-K^+^ but abnormal ECG-hypokalemia (ECG-K^+^ ≤3.5 mmol/L or false-positive hypokalemia) were more medically complex, including older age and more co-morbidities (DM, HTN, CKD, and HF), compared to those with normal Lab-K^+^ and ECG-K^+^ (3.6–5.4 mmol/L) (Fig. 5). Patients with ECG-dyskalemia but normal Lab-K^+^ also had significantly higher glucose, CRP, pBNP, D-dimer, proteinuria, and lower serum albumin. Patients with hypokalemic Lab-K^+^ but normal ECG-K^+^ (false-negative hypokalemia) were less medically complex, presenting with younger age, fewer co-morbidities (DM, HTN, CKD, HF, or proteinuria), lower stress markers (glucose, CRP, pBNP, D-dimer), and higher Alb than those with normal Lab-K^+^ and ECG-K^+^. This trend was less obvious in patients with hyperkalemic Lab-K^+^ but normal ECG-K^+^. Details of other characteristics are shown in Supplementary Fig. 6.

**Fig. 6:**
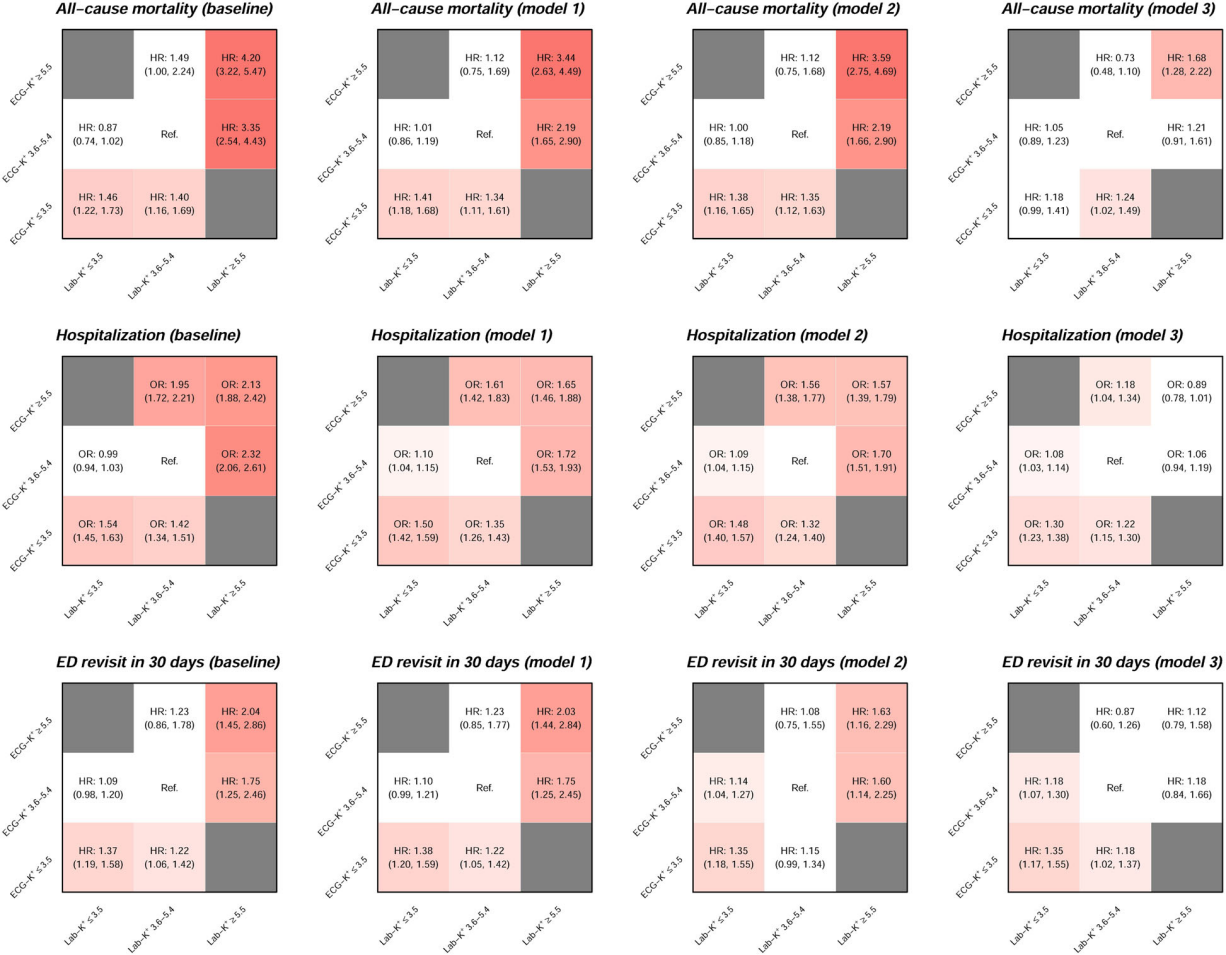
Risk matrices of different ECG-K^+^ and Lab-K^+^ groups on adverse outcomes in combined analysis. The baseline model of combined analysis is adjusted to each hospital site and based on Cox proportional hazard model or logistic regression as appropriate for each outcome. The color gradient represents the risk of the corresponding group and non-significant results are colored white. Model 1 includes significant demographic data (All-cause mortality: gender, Age, SBP, and DBP; Hospitalization: gender, age, BMI, DBP, and smoke; ED revisit in 30 days: gender). Model 2 includes the variables in model 1 and additional significant disease histories (All-cause mortality: HLP; Hospitalization: HLP, STK, and HF; ED revisit in 30 days: DM, CAD, STK, and COPD). Model 3 includes the variables in model 2 and additional significant laboratory tests (All-cause mortality: Hb, HCO_3_, Blood pH, Na, AST, ALT, Alb, CRP, pBNP, and D-dimer; Hospitalization: WBC, Hb, PLT, HCO_3_, PH, Na, Cl, tCa, GLU, AST, CK, Alb, CRP, TnI, and D-dimer; ED revisit in 30 days: Hb and Na).

Outcome analyses in patients with various combinations of Lab-K^+^ and ECG-K^+^ are shown in Fig. 6. Compared with patients with both normal Lab-K^+^ and ECG-K^+^ (3.6–5.4 mmol/L), those with concordant hyperkalemia (≥5.5 mmol/L by ECG and lab) and hypokalemia (≤3.5 mmol/L) had higher all-cause mortality within 30 days (HR for the former and the latter, 4.2 (95% CI 3.22–5.47) and 1.46 (95% CI 1.22–1.73), respectively). Patients with normal Lab-K^+^ but dyskalemic ECG-K^+^ exhibited higher HR for all-cause mortality in both extremes (HR 1.4, 95% CI 1.16–1.69 for hypokalemic ECG-K^+^ and HR 1.49, 95% CI 1.00–2.24 for hyperkalemic ECG-K^+^). However, the all-cause mortality risk were significantly lower in patients with abnormal Lab-K^+^ but normal ECG-K^+^ (HR 0.87 vs. 1.46 for the lab-hypokalemia group, and HR 3.35 vs. 4.2 for the lab-hyperkalemia group). In terms of hospitalization, the findings were similar to all-cause mortality. Patients with normal Lab-K^+^ but dyskalemic ECG-K^+^ had significantly higher risk for hospitalization [hypokalemic ECG-K^+^: odds ratio (OR) 1.42, 95% CI 1.34–1.51 and hyperkalemic ECG-K^+^: OR 1.95, 95% CI 1.72–2.21]. Similar trends were found regarding hypokalemic ECG-K^+^ on ED revisits, which persisted in adjusted models.

Based on the risk contribution from patients with discordant Lab-K^+^ and ECG-K^+^, we analyzed the added effect of ECG-K^+^ on each outcome. Compared to Lab-K^+^ alone, the integration of ECG-K^+^ with Lab-K^+^ yielded higher C-indices on all-cause mortality (C-index 0.634 vs. 0.598, *p* < 0.001), hospitalization (AUC 0.612 vs. 0.579, *p* < 0.001), and ED revisits (C-index 0.585 vs. 0.582, *p* < 0.001) (Supplementary Fig. 7). In fully-adjusted risk prediction models incorporating patient demographic, medical history, and other lab data, the addition of ECG-K^+^ further improved predictions for hospitalization but not all-cause mortality or ED revisits.

### ECG morphology and risk analysis in patients with discordant Lab-K^+^ and ECG-K^+^

**Fig. 7:**
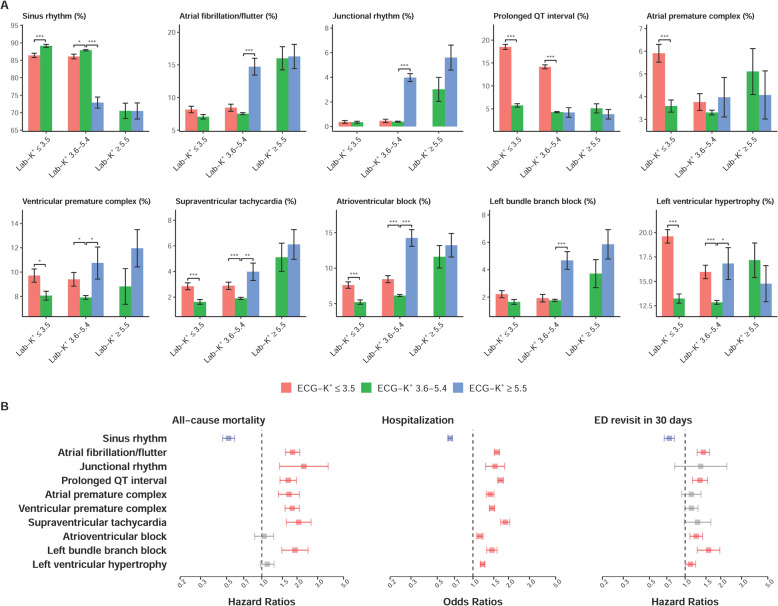
ECG morphology analysis of combinations of ECG-K^+^ and Lab-K^+^ on adverse outcomes. **A** Distributions of ECG morphology in each ECG-K^+^ and Lab-K^+^ group. Bars represent the mean or prevalence where appropriate and corresponding 95% conference intervals, which are adjusted by hospital. **B** Risk analysis of selected ECG morphologies on adverse outcomes. The hazard ratios and odds ratios were adjusted by hospital. Red, gray, and blue bars denote significantly positive, non-significant, and negative associations, respectively, with the corresponding outcomes.

Patients with normal Lab-K^+^ but abnormal ECG-hyperkalemia (ECG-K^+^ ≥5.5 mmol/L or false-positive hyperkalemia) had lower prevalence of sinus rhythm and higher prevalence of atrial fibrillation/flutter, junctional rhythm, premature ventricular complex, supraventricular tachycardia, atrioventricular block, left bundle branch block, and left ventricular hypertrophy, compared to those with normal Lab-K^+^ and ECG-K^+^ (3.6–5.4 mmol/L) (Fig. 7A). The false-positive hypokalemia group (ECG-K^+^ ≤3.5 mmol/L and 3.5 < Lab-K^+^ < 5.5 mmol/L) also had lower prevalence of sinus rhythm and higher prevalence of prolonged QT interval, premature ventricular complex, supraventricular tachycardia, atrioventricular block, and left ventricular hypertrophy, compared to those with normal Lab-K^+^ and ECG-K^+^ (3.6–5.4 mmol/L). Figure 7B shows the protective association of sinus rhythm and detrimental association of other morphologies on adverse outcomes.

### Sensitivity analysis

As shown in Supplementary Fig. 8, model performances were similar before and after the COVID-19 pandemic except for severe hypokalemia detection (K^+^ ≤ 2.5 vs. K^+^ > 2.5) at the community hospital due to the small sample size before the pandemic began (*n* = 6 vs. *n* = 3,134). Moreover, risk curves were similar between included (ECG and lab <1 h apart) and excluded patients (ECG and lab >1 h apart) for all-cause mortality and ED revisits (Supplementary Fig. 9) although the risk curve was significantly less pronounced for hospitalization. This was likely due to these patients having received treatment in the ED.

### Illustrations of patients with discordant Lab-K^+^ and ECG-K^+^

Supplementary Figs. 10–13 present ECGs from four patients with discordant Lab-K^+^ and ECG-K^+^. In each case, the AI model detected multiple concerning features and predicted an abnormal ECG-K^+^. These four patients all suffered poor outcomes despite their initially normal Lab-K^+^.

## Discussion

In this retrospective cohort study, we evaluated the diagnostic accuracy of AI-ECG for dyskalemias and clinical outcomes in >30,000 patients with both ECG-K^+^ and Lab-K^+^ data available. We found that AI-enabled ECG identified moderate-to-severe dyskalemia with good performance and faster than the laboratory. Stratified analyses showed that mild hypokalemia was called more often by ECG in the elderly with more co-morbidities. The U-shaped relationships between K^+^ concentration and adverse outcomes were more prominent for ECG-K^+^ than for Lab-K^+^. The HRs were high, with at least 4-fold increase in the risk for all-cause mortality among patients with both hyperkalemic ECG-K^+^ and Lab-K^+^. Compared to hypokalemic Lab-K^+^, patients with hypokalemic ECG-K^+^ had significantly higher risk for all-cause mortality, hospitalization and ED revisits after full confounder adjustment. Patients with normal Lab-K^+^ but abnormal ECG-K^+^ also exhibited more co-morbidities and incurred worse outcomes. The addition of ECG-K^+^ to Lab-K^+^ generally achieved better outcome prediction.

ECGs are commonly used as point-of-care (POC) tests to measure the electrical activity of the heart. Certain electrical changes in the ECG have been associated with dyskalemia, which can be confirmed by laboratory examination^8^. However, prompt recognition of dyskalemia-associated ECG changes prior to laboratory results is still fraught with great challenge in emergent situations. With advanced AI techniques^9^, ECG-based DLMs using large datasets of annotated ECGs have been developed that learn useful and subtle features over what is possible with manual interpretation^6,10,22^. In this retrospective study, longer ECG signals (10 s vs. 2.5 s) were found to achieve better diagnostic accuracy with lower MAE (0.365 vs. 0.531 mmol/L) than other recently-published retrospective studies^6,23^. The early or almost simultaneous collection of ECG with bloodwork reduced the chance of Lab-K^+^ biased by treatment, in stark contrast to other reports in which ECGs were obtained 1 to 6 h before or after blood tests. As expected, the complete 12-lead analysis performed better than any single lead. Of note, the present AUCs of 0.932-0.942 for detecting Lab-K^+^ ≥5.5 mmol/L were higher than others using 2 to 4 leads (AUCs 0.853-0.901)^10^.

In our ED patients, the prevalence of hyperkalemia was approximately 2%, which is in line with other reports of hyperkalemia occurring in 2–5% of ED patients^5^. Despite AUC greater than 0.93 for hyperkalemia detection, the positive predictive value is low (10.4–14.7%) in the setting of low prevalence of hyperkalemia. Although this seemingly high false positivity rate of AI-ECG for hyperkalemia may cause anxiety and inconvenience for clinicians and patients, pseudo-positive EKG-K^+^ for hyperkalemia, importantly, predicts adverse outcomes due to ECG changes directly reflecting underlying cardiac and non-cardiac disorders. This finding is similar to the “previvor” patients in a recent study of AI-enabled ECG analysis for left ventricular systolic dysfunction^24^, which showed that those individuals with apparently false-positive AI-ECG findings had a fourfold increased risk of developing ventricular dysfunction over the ensuing 5 years^14^.

Both hyperkalemia and hypokalemia are associated with significant morbidity and mortality. The U-shaped association between dyskalemic Lab-K^+^ and mortality has been well documented in patients with different diseases such as DM, CKD, AMI, and HF. For example, hyperkalemia, commonly seen in patients with CKD and HF, is an independent predictor for mortality^19^ and also confer a higher risk of cardiovascular events and hospitalization^25^. Hyperkalemia per se can reduce heart rate, cardiac function, and induce severe arrhythmias as well as circulatory collapse^26–29^. In this study, we evaluated the outcomes associated with laboratory dyskalemia in ED patients with different underlying diseases and acute illnesses. We found that patients with hyperkalemia had higher mortality, hospitalization, and ED revisits compared to those with normokalemia. However, patients with hypokalemia did not have significantly higher mortality despite higher hospitalization rate and ED revisits. This finding was similar to those of some prior reports^5^ but not others^3,4^. In fact, the association between laboratory hypokalemia and mortality remains controversial, depending on the study design and different studied populations. Hypokalemia directly affects cardiac function, induces arrhythmias, and indirectly serves as a surrogate marker for the severity of underlying diseases, such as CHF, AMI, and even CKD^19,28^. At the same time, hypokalemia in patients with acute illnesses (frequently seen in the ED) can also be an epiphenomenon for stress and enhanced sympathetic activity, causing acute K^+^ shift into cells. Our null finding on a hard outcome (mortality) but positive findings on outcomes subject to physician and patient interpretation (hospitalization and ED revisits), suggest that laboratory hypokalemia may often be a marker for severe stress.

This study investigated the association between abnormal ECG-K^+^ and outcomes in ED patients. Overlapping curves for mortality, hospitalization, and ED revisits were observed for patients with hyperkalemic ECG-K^+^ and Lab-K^+^ but not for those with hypokalemic ECG-K^+^ and Lab-K. In contrast to no association for laboratory hypokalemia, hypokalemic ECG-K^+^ was associated with a 1.5-fold increased risk for mortality. There are several important differences between hypokalemic Lab-K^+^ and ECG-K^+^. The prevalence of ECG-K^+^ of ≤3.5 mmol/L was lower than Lab-K^+^ ≤3.5 mmol/L and the ECG-K^+^ was relatively less sensitive for detecting mild to moderate lab-hypokalemia. However, we found that patients with hypokalemic ECG-K^+^ were more medically complex than those with hypokalemic Lab-K^+^. Our stratified analysis for detecting mild ECG-K^+^ hypokalemia showed the intensity of association became significantly higher in the elderly with more co-morbidities. Conversely, patients with normal ECG-K^+^ but hypokalemic Lab-K^+^ were less likely to be elderly and in better physical condition. Typically, less than 50% of hypokalemic patients exhibit visible ECG changes^30^, implying the ECG changes picked up by ECG-K^+^ mark pathophysiology beyond just an abnormal electrolyte. These findings suggest that hypokalemic ECG-K^+^ may be thought of like a cardiac troponin, a reflection of myocardial injury which may be caused by a variety of cardiac and non-cardiac etiologies and portending worse outcomes.

Further evidence of this comes from patients with normal Lab-K^+^ but abnormal ECG-K^+^ (detection error by ECG-K^+^), who had older age, more co-morbidities (DM, HTN, CKD, and HF), and multiple laboratory abnormalities. They clearly demonstrated adverse outcomes with higher HR for all-cause mortality (HR 1.40 and 1.49 for hypokalemic and hyperkalemic ECG-K^+^, respectively). In contrast, false-negative patients for ECG-K^+^ detection (normal ECG-K^+^ but dyskalemic Lab-K^+^) had significantly lower risk for all-cause mortality (HR = 0.87 vs. HR = 1.46 for hypokalemia and HR = 3.35 vs. HR = 4.20 for hyperkalemia). Why the ECG-K^+^ encodes additional prognostic information beyond the electrolyte value is an interesting question. Another group has shown that a DLM could be trained to detect systolic dysfunction using the ECG^14^. Patients with hypo- and hyperkalemia were enriched with conditions such as DM, AMI, CKD and HF compared to patients with normokalemia. In the training process for our DLM, besides learning the ECG differences between normal and dyskalemias, it also learned the subtle ECG differences ascribed to diseases that contribute to K^+^ abnormalities. Hence, discrepant ECG-K^+^ and Lab-K^+^ could be a useful signal to treating physicians to be more careful and cast a larger differential when treating those patients.

There are several clinical applications to using ECG-K^+^ to detect dyskalemia. ECG is a simple, cheap, and non-invasive test to provide specific K^+^ value with similar timeliness to point-of-care (POC) tests. The very good accuracy of our ECG-K^+^ means a background monitoring system could be implemented in the ED to actively calculate ECG-K^+^ for every ECG, not limited to acute myocardial infarction and atrial fibrillation. It has been reported that patients with severe dyskalemia may develop sudden fatal cardiac arrhythmias within 1 h of triage—prior to the laboratory report^31–33^. The ~40 min lead time afforded by ECG-K^+^ means severe dyskalemias can be prioritized on the differential, potentially life-saving interventions readied at the bedside, and given immediately upon receipt of a concordant lab result. If the results are discordant, these are still opportunities for physicians to think more carefully about the patients’ underlying conditions due to the higher mortality and other adverse outcomes.

There were some limitations of this study. First, although our study demonstrated that ECG-K^+^ was quicker than the central lab, we did not evaluate early interventions based on the ECG-K^+^. Second, our patient population is East Asian and ECG characteristics may vary by race although the diagnostic performance of DLM may still be stable, especially within the same population^12^. An international study involving different racial and ethnic groups should be conducted to validate the performance of ECG-K^+^ in different settings. Third, patients may have visited other EDs or expired at other hospitals, which would not be captured in our EMR. We believe the likelihood of incomplete capture is low as a previous study of readmissions in Taiwan using the government’s National Health Insurance Research Database found only 0.16% of readmissions occurred at a different hospital^34^. Fourth, the “black box” of DLM necessitates our ECG-K^+^ being more transparent^35^. Given the relatively poor discriminative ability of well-known ECG changes in dyskalemias, further exploration of other relevant ECG morphologies is needed. Finally, POC-K^+^ has been used to detect dyskalemias early. We did not compare the accuracy of ECG-K^+^ to POC-K^+^.

In conclusion, this multi-site retrospective study not only validated the accuracy of ECG-K^+^, especially in moderate-to-severe dyskalemia, but also revealed meaningful differences between ECG-K^+^ and Lab-K^+^. The ECG-K^+^ holds promise as a biomarker for adverse ED outcomes. Before clinicians take advantage of AI-ECG-K to rapidly diagnose and support treatment decisions for patients with severe dyskalemia, a large-scale, international trial with ECG-K^+^-based intervention is needed to validate its clinical application.

## Method

### Ethical statement

This study was approved by the institutional review board of Tri-Service General Hospital, Taipei, Taiwan (IRB NO. C202005055). Patients’ consent was waived because data were collected retrospectively and in anonymized files and encrypted from the hospital to the data controller.

### Study design and population

We performed a black-box pragmatic study in the EDs of two hospitals within the Tri-Service General Hospital health care system between May 1, 2019 and December 31, 2020. Data were transmitted in real time to an integration engine and database server where the ECG-K^+^ were calculated and stored without display to clinical providers. The hospital electronic medical record system was re-designed to maximize data completeness in this study. Where possible, all relevant data items were changed to required fields to reduce missing values for this study. The first site was an academic medical center (NeiHu General Hospital) with 1,800 beds and accommodating 100,000 ED visits annually before the COVID-19 pandemic. The second site was a community hospital (Tingzhou Branch Hospital) with 100 beds and accommodating 15,000 ED visits annually before the pandemic. Because the COVID-19 pandemic was in study period, we conducted a sensitivity analysis to explore its potential impact on the accuracy of ECG-K^+^ due to a shift to fewer low-acuity patients in the ED^36^.

The algorithm for collecting patient data with ED visits at both hospitals are shown in Fig. 1A. There were 50,358 and 8223 ED visits to the participating medical center and community hospital, respectively. All ED visits started with an initial assessment at the triage station. Those whose interval between ECG and Lab-K^+^ measurement was longer than 1 h were also excluded, due to the possibility of having received treatments which alter the ECG during the long time interval. In total, 34,803 ED visits from 26,499 patients to the medical center and 6492 visits from 4747 patients to the community hospital were recruited for analysis. A sensitivity analysis to compare outcome predictions between included and excluded patients was conducted.

### Measurement of ECG-K^+^ and Lab-K^+^

ECGs were obtained when patients were supine, using a standard 10 s, 12-Lead Philips ECG machine (PH080A). The ECG-K^+^ ranged from 1.5 mmol/L to 7.5 mmol/L, estimated by ECG12Net, a DLM with 82 convolutional layers developed previously. This system is configured to visualize the basis for the AI predictions using class activation mappings and attention mechanism^6^. We used complete signals of 10 seconds instead of the conventional 2.5 s. Nine overlapping sequences of length 1024 were used to generate predictions and averaged to yield the final prediction as previously described^16,37–39^.

Lab-K^+^ determination was based on central laboratory methods. Laboratory peudo-dyskalemia were excluded based on evidence of hemolysis and plasma K^+^ interference indices. We divided the data into seven categories based on Lab-K^+^ concentrations: severe hypokalemia (≤2.5 mmol/L), moderate hypokalemia (2.5 < Lab-K^+^ ≤ 3.0 mmol/L), mild hypokalemia (3.0 < Lab-K^+^ ≤ 3.5 mmol/L), normal (3.5 < Lab-K^+^ < 5.5 mmol/L), mild hyperkalemia (5.5 ≤ Lab-K^+^ < 6.0 mmol/L), moderate hyperkalemia (6.0 ≤ Lab-K^+^ < 6.5 mmol/L), and severe hyperkalemia (≥6.5 mmol/L). The same classification was applied to ECG-K^+^.

### Study covariates

Study covariates, including demographics, medical co-morbidities, and laboratory tests, were obtained from the electronic medical record. We used International Classification of Diseases, Ninth Revision and Tenth Revision to define diabetes mellitus (DM), hypertension (HTN), hyperlipidemia (HLP), chronic kidney disease (CKD), coronary artery disease (CAD), stroke (STK), heart failure (HF), and chronic obstruction pulmonary disease (COPD). In addition to Lab-K^+^, we also collected other laboratory values in the ED, including complete blood cell count, blood pH, bicarbonate (HCO_3_), electrolytes, liver and renal function profiles, glucose (GLU), creatine kinase (CK), albumin (Alb), C-reactive protein (CRP), procalcitonin (PCT), troponin I (TnI), NT-pro-B type natriuretic peptide (pBNP), D-dimer, and urine protein. Missing data were imputed using multiple imputations in multivariate analysis^40^. We selected certain important ECG morphology based on the structured findings statements that are standard on the Philips system. These included sinus rhythm, atrial fibrillation/flutter, junctional rhythm, prolonged QT interval, atrial premature complex, ventricular premature complex, supraventricular tachycardia, atrioventricular block, left bundle branch block, and left ventricular hypertrophy.

### Outcome variables

The outcomes of interest included all-cause mortality, hospitalization, and ED revisit. For mortality, the survival time was calculated with reference to the date of the ED visit. Patient status (dead/alive) was captured through the electronic medical record. Moreover, data for alive visits were censored at the patient’s last known hospital alive encounter to limit bias from incomplete records. The end of follow-up in this study was January 31, 2021.

For hospitalizations, each ED visit was assigned a disposition at discharge, including mortality, admission, and discharge home. For ED revisits, we analyzed the discharged patients for revisits within 30 days. Patients with ED revisit or re-admission were assigned as positive events. We calculated the follow-up duration from the original date of ED visit. Negative samples were censored at the patient’s last known hospital activity and the end of follow-up was January 31, 2021.

### Statistical analysis

This study included two parts, the accuracy analysis and outcome analysis. All statistical analyses were done by R version 3.4.4. The significance level was set at *p* < 0.05. For the accuracy analysis, we primarily focused on the discrimination between different severities of hypo/hyperkalemia. The ECG-K^+^ was used to generate receiver operating characteristic curves and AUC to evaluate performance. Pearson correlation coefficients (COR) and MAE were used to demonstrate the relationship between ECG-K^+^ and Lab-K^+^.

Outcome analysis used subjects from both study sites to expand sample size. We also adjusted for the hospital site as a confounder. We first explored differences in characteristics of each ECG-K^+^ group sharing the same Lab-K^+^. Linear or logistic regression were used for statistical analyses where appropriate. Cox proportional hazard model was used to analyze the impact of ECG-K^+^, Lab-K^+^, and study covariates on outcomes of interest. We used the “pspline” function with a degree of freedom of 2 in the “survival” package to examine the U-shaped relationship in analyses involving ECG-K^+^ and Lab-K^+^. The other variables were used as linear predictors in a stepwise program based on significant tests. The significance levels of forward and backward regressions were set at 0.0001 and 0.001 based on Bonferroni correction, respectively. We divided these selected variables into three categories (demographic profile, disease history, and laboratory test) for hierarchical adjustment. Hazard ratios (HRs) and 95% conference intervals (CIs) were computed for risk estimation of each group. A series of integration models were evaluated using the C-index the marker of global performance to explore the additional contribution of ECG-K^+^.

### Reporting summary

Further information on research design is available in the Nature Research Reporting Summary linked to this article.

## Supplementary information

Supplementary Information

Reporting Summary

## Data Availability

The data analyzed in this study is not publicly available due to privacy and security concerns. The data may be shared with a third party upon execution of data sharing agreement for reasonable requests, such requests should be addressed to C.L. (e-mail: xup6fup@mail.ndmctsgh.edu.tw) or S.H.L.

## Code availability

The code may be made available from the authors upon reasonable request subject to permission and approval from the corresponding organizations and institutional review boards.
