# Supplementary material for: Point-of-care artificial intelligence-enabled ECG for dyskalemia: a retrospective cohort analysis for accuracy and outcome prediction
Source: NPJ Digit Med. 2022 Jan 19;5:8. doi: 10.1038/s41746-021-00550-0 (PMC8770475; doi:10.1038/s41746-021-00550-0)
Supplement: Supplementary file 1 — Supplementary Information [file 41746_2021_550_MOESM1_ESM.pdf]

Supplementary Table 1 | ED visit based patient characteristics at an academic medical center and a community hospital.

	Academic medical center (Neihu site) 34,803 visits | 26,499 patients	Community hospital (Tingzhou site) 6,492 visits | 4,747 patients	p-value
**K^+ (mmol/L)**	3.9±0.6	4.0±0.5	0.026
**K^+ group**			<0.001
K^+ ≤ 2.5	120 (0.3%)	15 (0.2%)	
2.5 < K^+ ≤ 3.0	921 (2.6%)	121 (1.9%)	
3.0 < K^+ ≤ 3.5	6,272 (18.0%)	1,015 (15.6%)	
3.5 < K^+ < 5.5	26,745 (76.8%)	5,249 (80.9%)	
5.5 ≤ K^+ < 6.0	412 (1.2%)	53 (0.8%)	
6.0 ≤ K^+ < 6.5	156 (0.4%)	27 (0.4%)	
6.5 ≤ K^+	177 (0.5%)	12 (0.2%)	
**Demography**			
Gender (male)	17,570 (50.5%)	3,075 (47.4%)	<0.001
Age (years)	61.3±19.5	65.2±21.3	<0.001
BMI (kg/m^2)	24.2±4.3	24.1±4.1	0.036
SBP (mmHg)	137.3±27.7	141.9±28.2	<0.001
DBP (mmHg)	78.9±17.4	74.0±17.4	<0.001
Smoking/Ever	9,745(28.0%)	1,796(27.7%)	0.580
**Disease history**			
DM	8,664(24.9%)	2,109(32.5%)	<0.001
HTN	13,623 (39.1%)	3,337 (51.4%)	<0.001
HLP	11,065 (31.8%)	2,795 (43.1%)	<0.001
CKD	5,188(14.9%)	990(15.2%)	0.477
CAD	8,941 (25.7%)	2,077 (32.0%)	<0.001
STK	6,374 (18.3%)	1,623 (25.0%)	<0.001
HF	4,235(12.2%)	970(14.9%)	<0.001
COPD	5,429(15.6%)	1,793(27.6%)	<0.001
**Laboratory test**			
WBC (10^3/ul)	8.9±5.9	8.8±4.8	0.096
Hb (gm/dL)	12.6±2.4	12.8±2.3	<0.001
PLT (10^3/ul)	234.2±87.6	223.2±78.2	<0.001
Blood pH	7.4±0.1	7.4±0.1	0.022
HCO_3 (mmol/L)	24.1±4.0	24.4±3.9	<0.001
Na^+ (mmol/L)	136.9±4.6	137.4±4.6	<0.001
Cl^- (mmol/L)	103.8±5.5	104.2±5.3	<0.001
tCa^{++} (mg/dL)	8.6±0.7	8.6±0.7	0.471
GLU (gm/dL)	142.1±76.9	141.6±69.6	0.611
AST (U/L)	29.8±66.9	27.6±61.1	0.013
ALT (U/L)	26.0±65.4	24.4±55.6	0.066
CK (U/L)	151.1±561.3	116.7±184.6	<0.001
Cr (mg/dL)	1.6±2.1	1.3±1.5	<0.001
BUN (mg/dL)	23.7±22.4	22.4±19.5	<0.001
Alb (g/dL)	3.6±0.6	3.6±0.5	0.460
CRP (mg/dL)	2.9±5.5	2.5±4.8	<0.001
PCT (ng/mL)	1.4±7.8	1.0±5.8	<0.001
TnI (ng/mL)	87.3±871.8	52.2±614.5	0.002
pBNP (pg/mL)	2,478.3±6,924.8	1,929.0±5,789.8	<0.001
D-dimer (ng/mL)	2,102.7±4,683.6	1,841.3±3,996.8	<0.001
Urine protein			0.004
-	10,853(59.9%)	2,059(57.2%)	
1+	4,301(23.7%)	882(24.5%)	
2+/3+/4+	2,973(16.4%)	660(18.3%)	

Abbreviations: ED, emergency department; K^+, potassium; BMI, body mass index; SBP, systolic blood pressure; DBP, diastolic blood pressure; DM, diabetes mellitus; HTN, hypertension; HLP, hyperlipidemia; CKD, chronic kidney disease; CAD, coronary artery disease; STK, stroke; HF, heart failure; COPD, chronic obstruction pulmonary disease; WBC, white blood cell count; Hb: hemoglobin; PLT, platelet; HCO_3, bicarbonate; Na^+, sodium; Cl^-, chloride; tCa^{++}, total calcium; AST, aspartate aminotransferase; GLU, glucose; ALT, alanine aminotransferase; CK, creatine kinase; Cr, Creatinine; BUN, blood urea nitrogen; Alb, albumin; CRP, C-reactive protein; PCT, procalcitonin; TnI, troponin I; pBNP, NT-pro-B type natriuretic peptide.

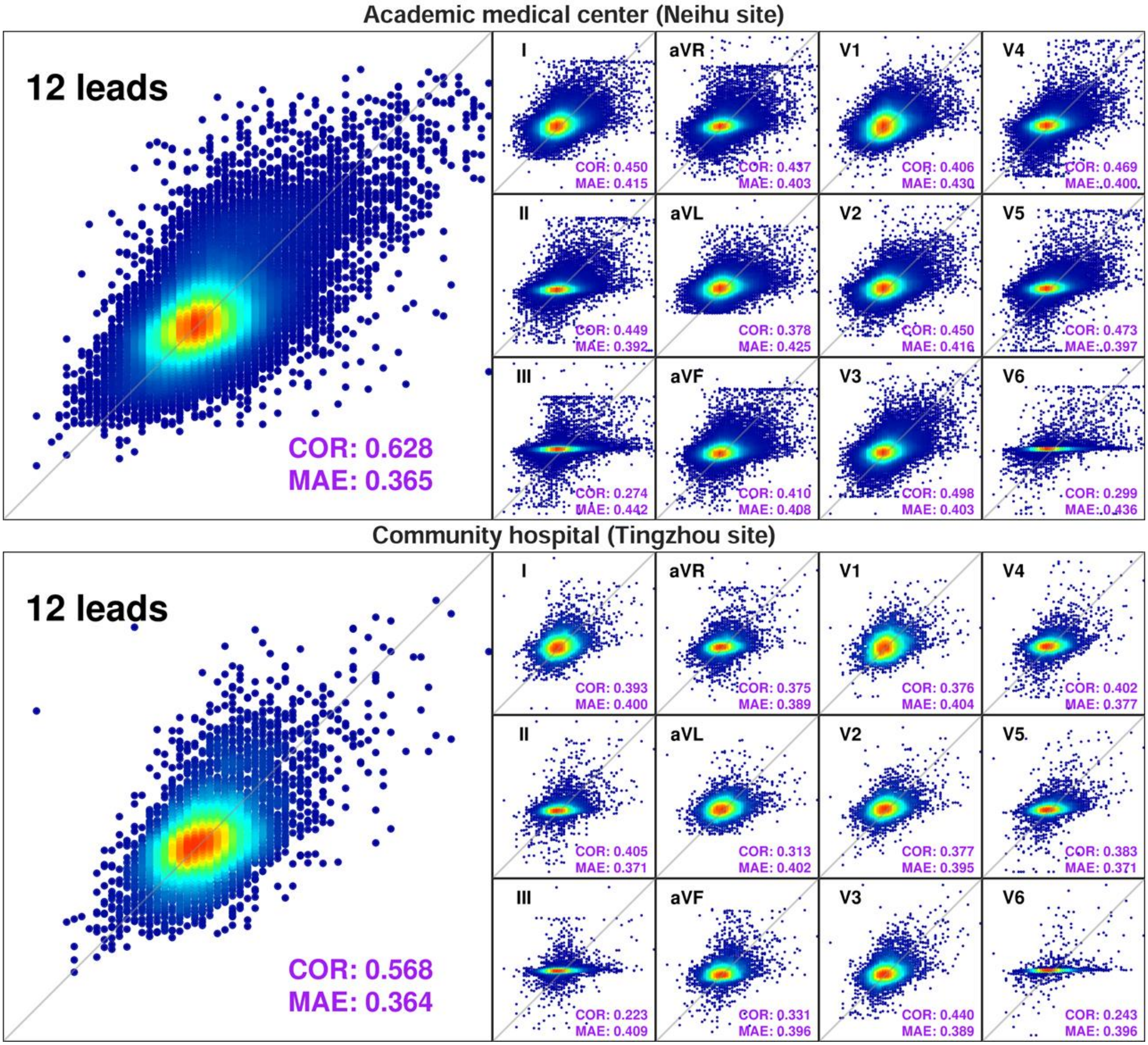

Supplementary Figure 1 | Scatter plots of ECG-K^+ and Lab-K^+ at an academic medical center and a community hospital. The x-axis indicates the Lab-K^+. The y-axis presents the ECG-K^+. Red points represent the highest density, followed by yellow, green light blue, and dark blue. Perfect model performance would fall only along the diagonal line. We presented the Pearson correlation coefficients (COR) and mean absolute errors (MAE) to compare ECG-K^+ estimated via information from 12 leads and each lead.

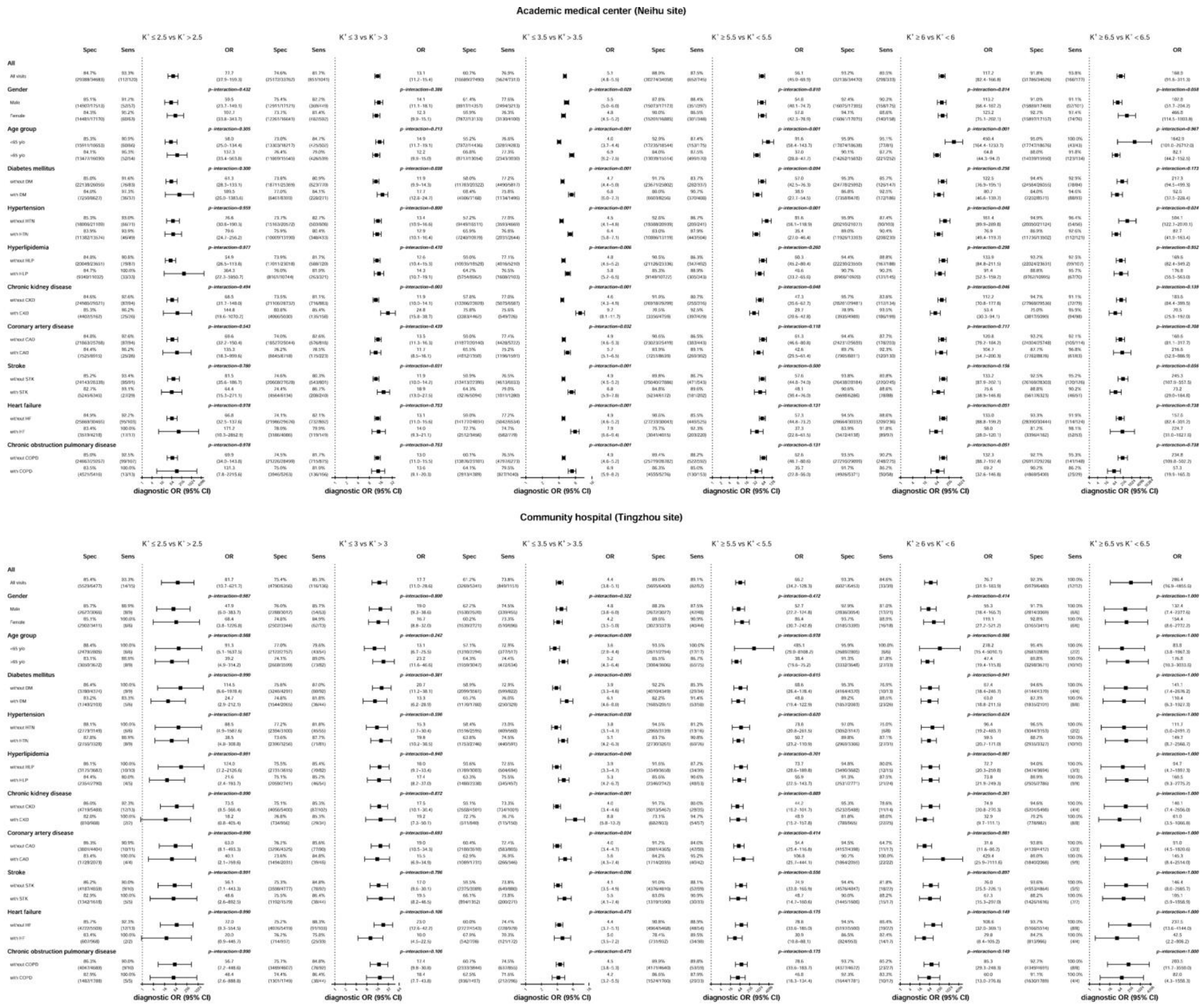

Supplementary Figure 2 | Stratified analyses for the performance of ECG-K$^+$ for detecting mild to severe hypo/hyper-kalemia. The sensitivity and specificity are tabulated across a series of stratified conditions. The diagnostic OR, which is the ratio of positive likelihood ratio (sensitivity/(1- specificity)) to the negative likelihood ratio ((1-sensitivity)/specificity), as well as the associated 95% CI, is shown for each situation. All analyses were checked for problematic zero counts, which were remedied by adding a fixed value of 0.5 to all cells where the problem occurred. The ECG-K$^+$ is estimated via information from 12 leads in this analysis. The p-interaction was the significance test of strength of association, with an adjusted significance level of 0.001 based on Bonferroni correction. Based on this correction, the only significant strength of association was for detecting mild hypokalemia ($p < 0.001$).

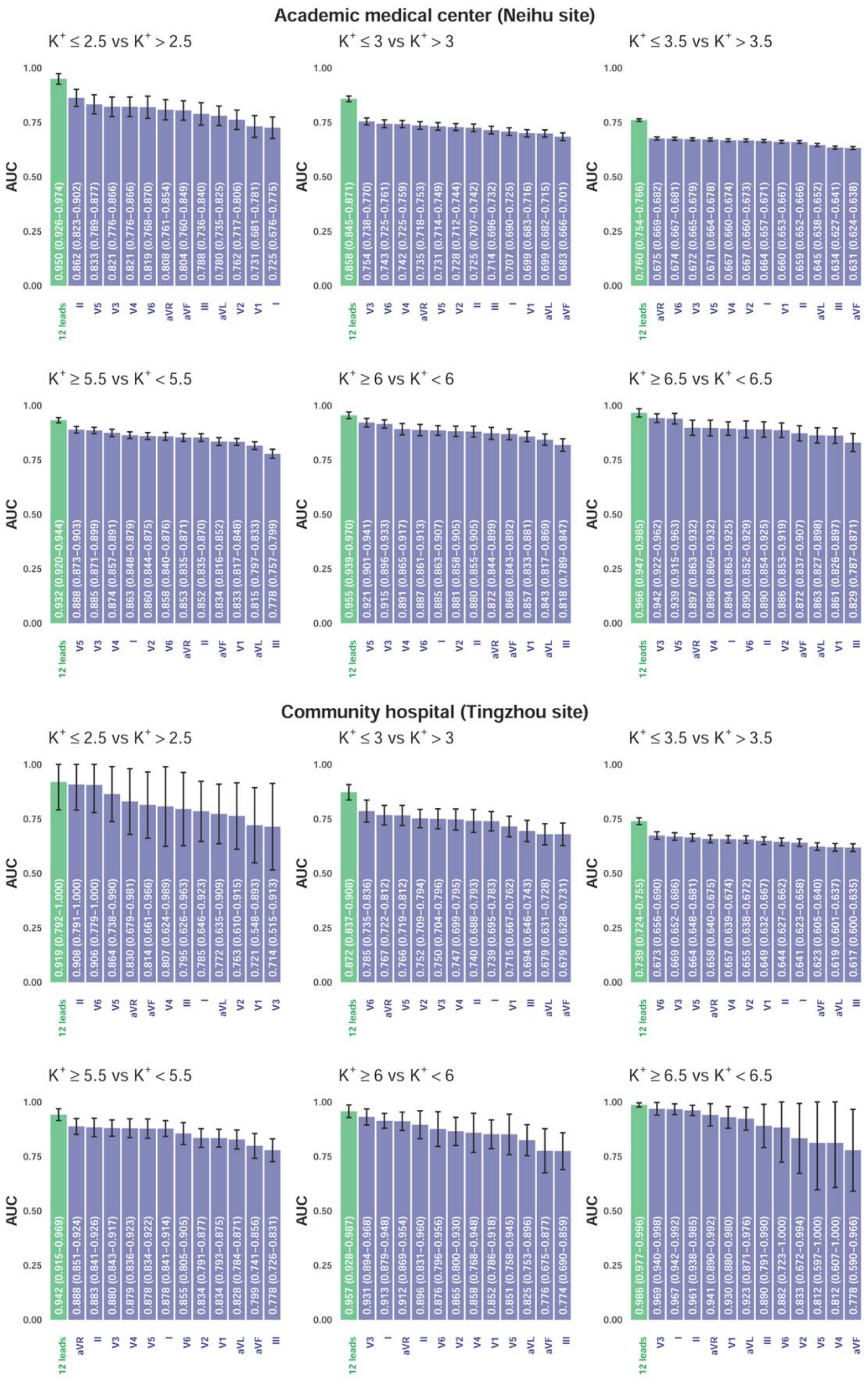

Supplementary Figure 3 | Performance ranking of ECG-K$^+$ via information from 12 leads and each lead individually for detecting mild to severe hypo/hyper-kalemia. All analyses were conducted for an academic medical center and a community hospital simultaneously. The y-axis presents the area under of receiver operating characteristic curve (AUC) based on the definitions of cases and controls in the subtitles.

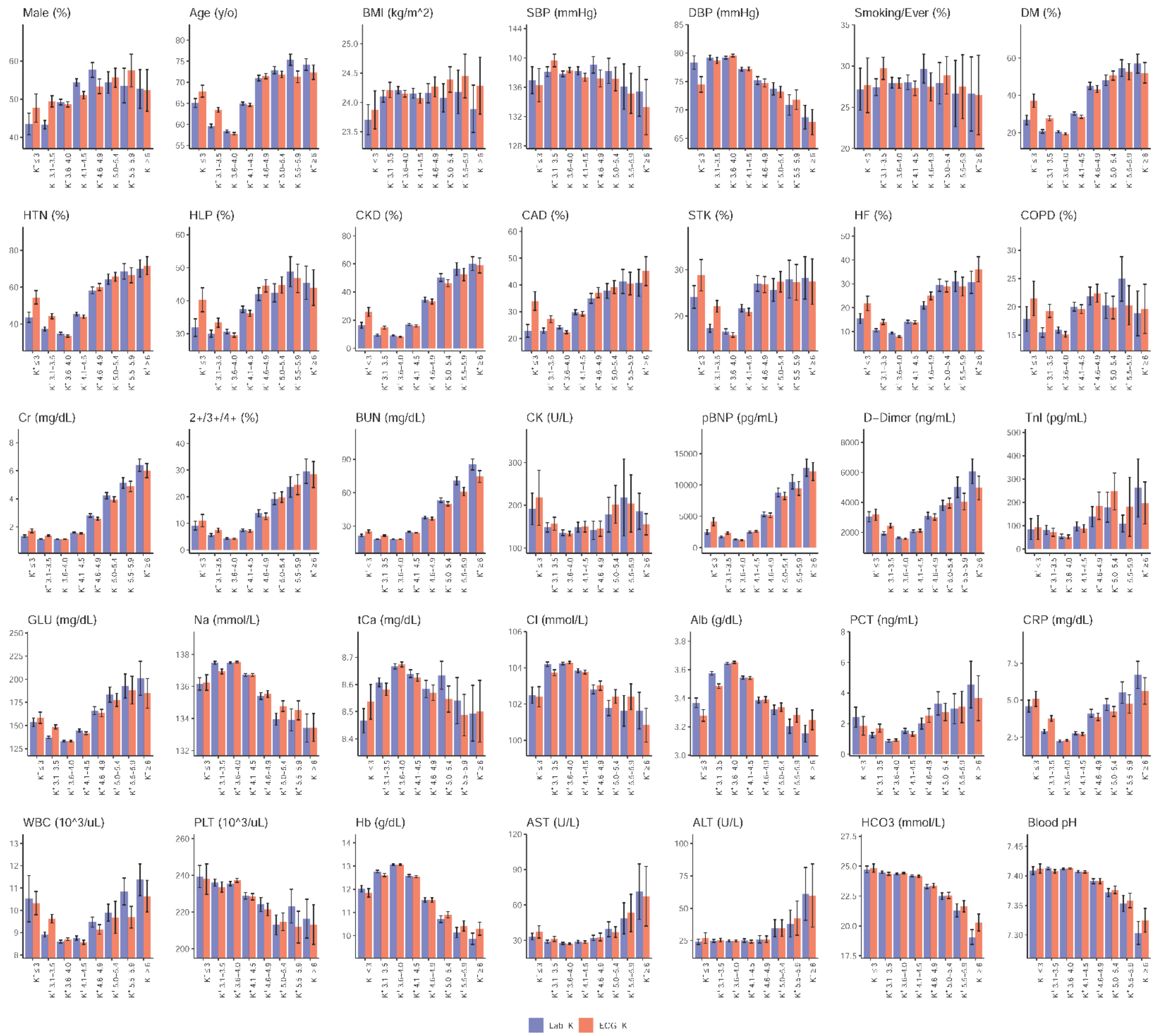

Supplementary Figure 4 | Distributions of patient characteristics in each ECG-K$^+$ and Lab-K$^+$ group. Bars represent the mean or proportion where appropriate and corresponding 95% conference intervals, which are adjusted by hospital.

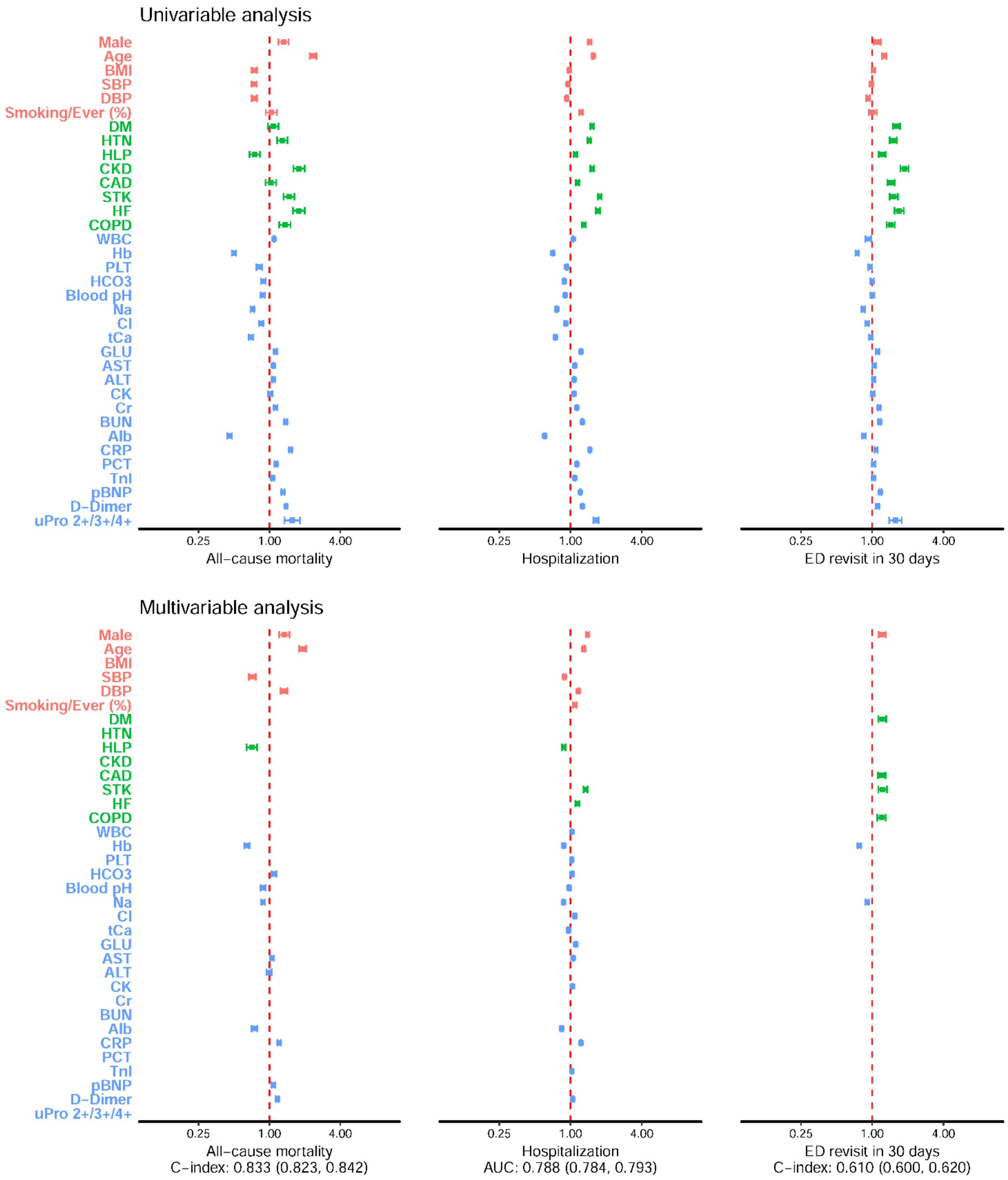

Supplementary Figure 5 | Risk effect analysis of patient characteristics on outcomes of interest. Univariable and multivariable analyses were conducted by Cox proportional hazard model and logistic regression, respectively. All analyses were adjusted by hospital site, including univariable analysis. Continuous variables are standardized by mean and standard deviation, so the units of each continuous variable were 1 standard deviation. The selected variables in multivariable analyses were based on stepwise process for each outcome.

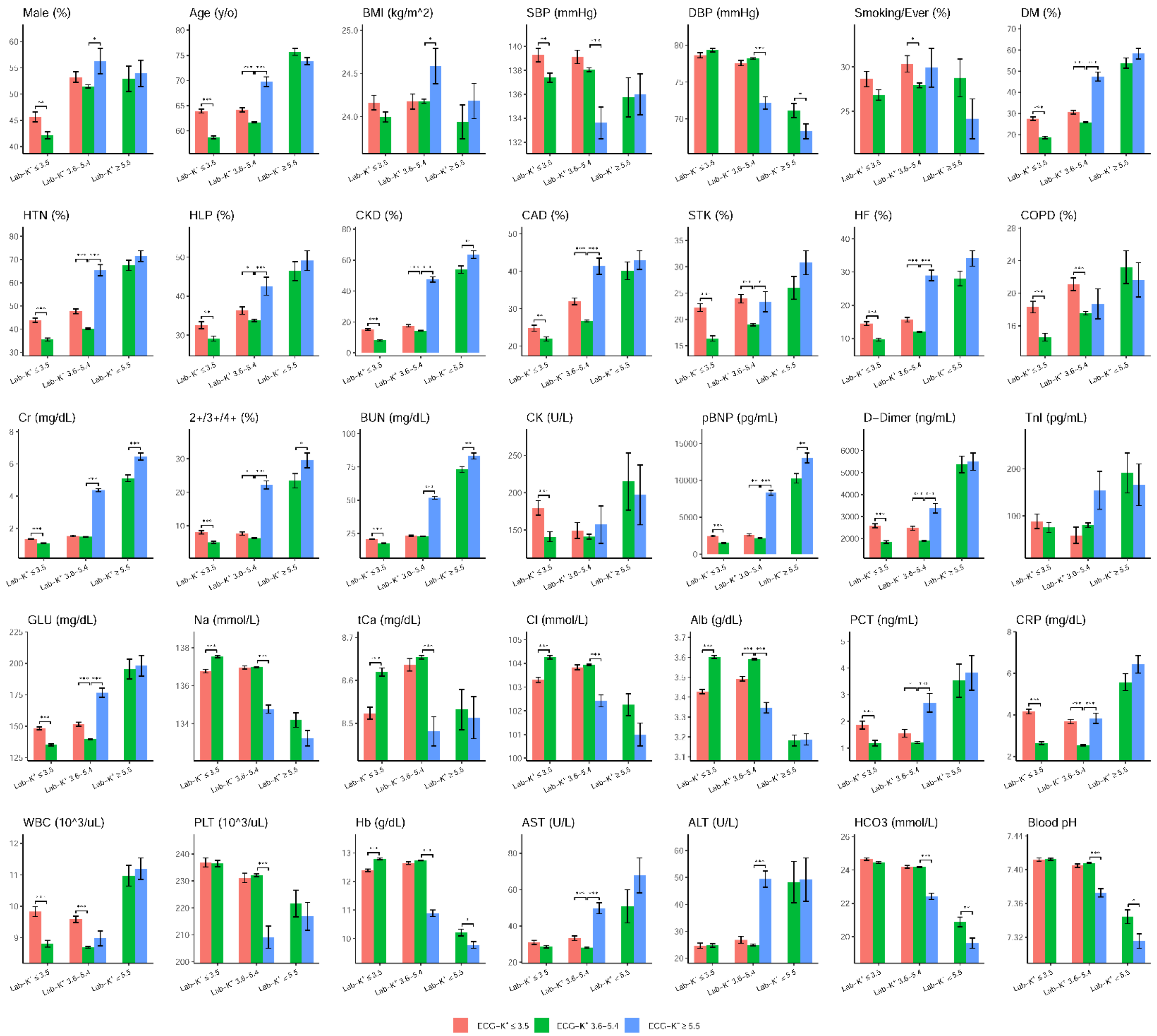

Supplementary Figure 6 | Patient characteristics in different ECG-K$^+$ groups and Lab-K$^+$ groups. Bars represent the mean or proportion where appropriate and corresponding 95% conference intervals, which are adjusted by hospital and Lab-K$^+$ via linear or logistic regression (*: p < 0.05; **: p < 0.01; ***: p < 0.001).

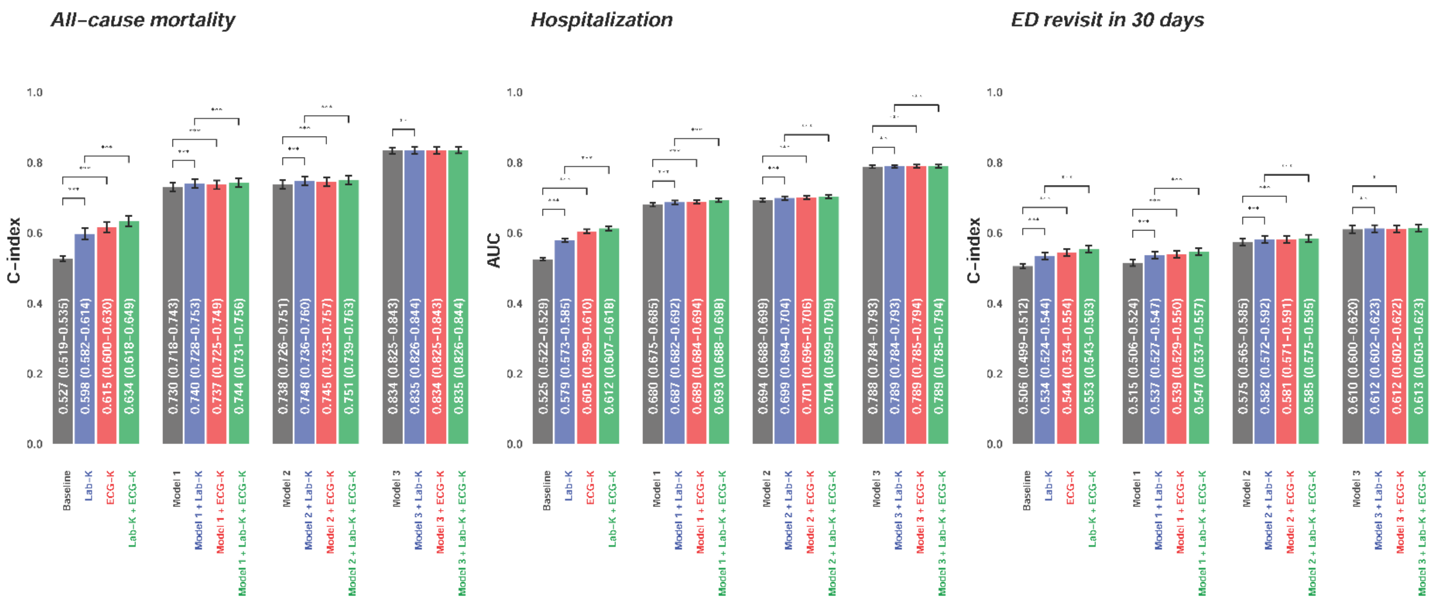

Supplementary Figure 7 | Risk contribution analysis of additional consideration of ECG-K$^+$. The baseline model of combined analysis is adjusted to each hospital site and based on Cox proportional hazard model or logistic regression as appropriate for each outcome. C-index and AUC are used as the performance assessment where appreciate. Model 1 includes significant demographic data (All-cause mortality: gender, Age, SBP, and DBP; Hospitalization: gender, age, BMI, DBP, and smoke; ED revisit in 30 days: gender). Model 2 includes the variables in model 1 and additional significant disease histories (All-cause mortality: HLP; Hospitalization: HLP, STK, and HF; ED revisit in 30 days: DM, CAD, STK, and COPD). Model 3 includes the variables in model 2 and additional significant laboratory tests (All-cause mortality: Hb, HCO_3, Blood pH, Na, AST, ALT, Alb, CRP, pBNP, and D-dimer; Hospitalization: WBC, Hb, PLT, HCO_3, PH, Na, Cl, tCa, GLU, AST, CK, Alb, CRP, TnI, and D-dimer; ED revisit in 30 days: Hb and Na). Abbreviations: *, $p < 0.05$; **, $p < 0.01$; ***, $p < 0.001$.

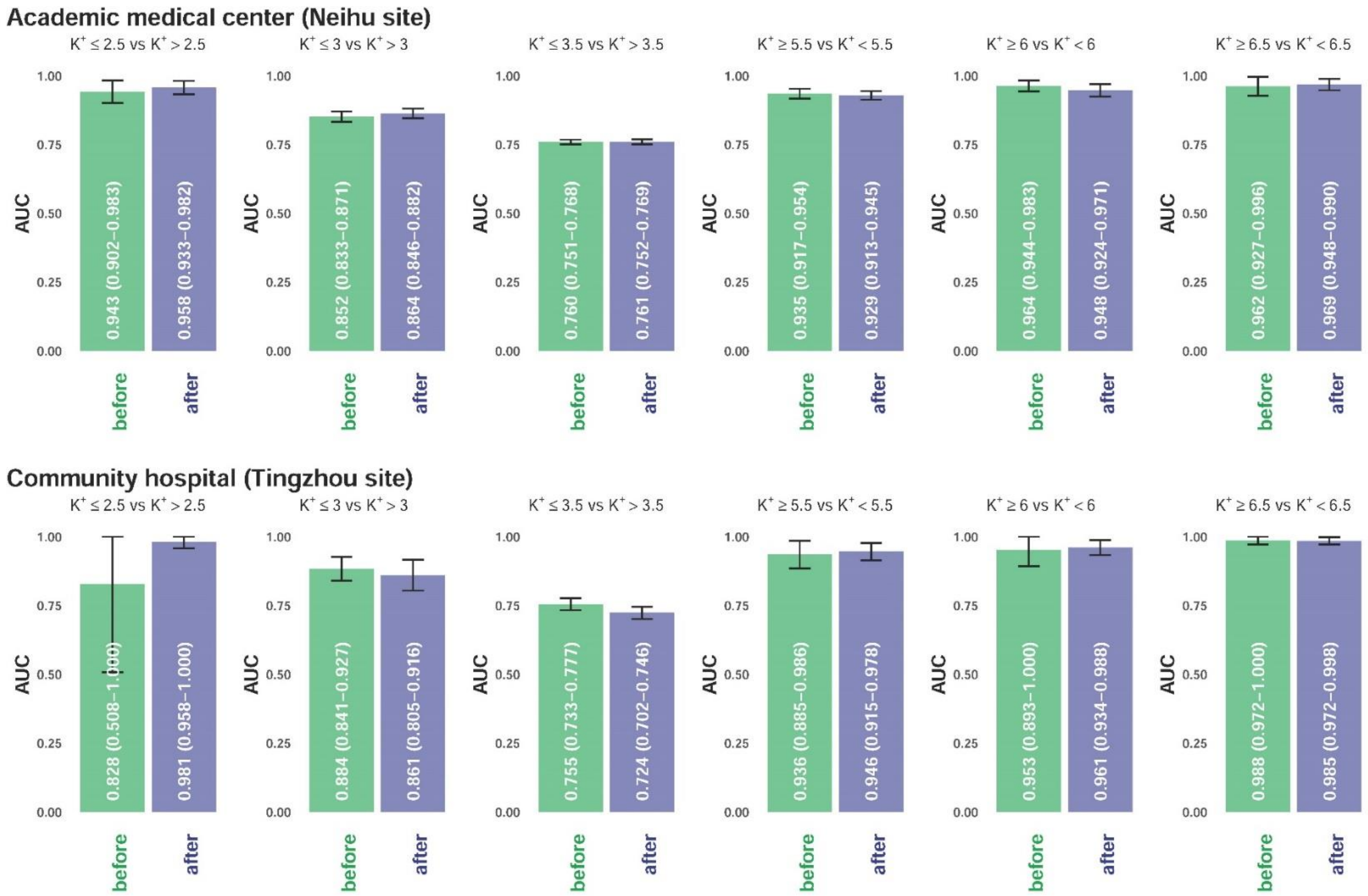

Supplementary Figure 8 | Performance comparison before and after COVID-19 pandemic for detecting mild to severe hypo/hyper-kalemia. All analyses were conducted for an academic medical center and a community hospital simultaneously. The y-axis presents the area under of receiver operating characteristic curve (AUC) based on the definitions of cases and controls in the subtitles. We used February 6, 2020 to distinguish the start of the pandemic period as fever screening stations began on that date per the central preventive policy in Taiwan.

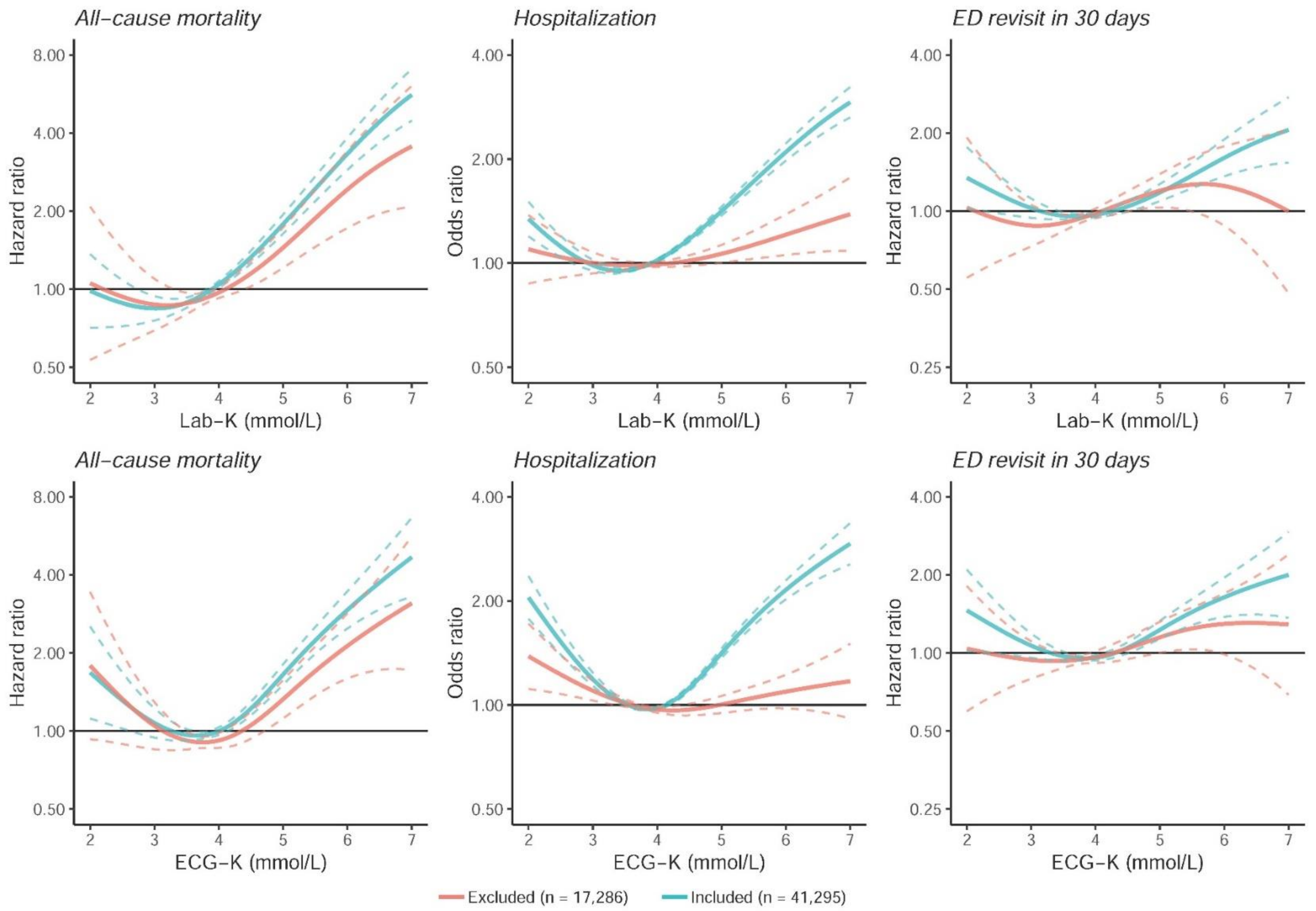

Supplementary Figure 9 | Continuous association of ECG-K$^+$ and Lab-K$^+$ on adverse outcomes in included and excluded patients. The upper and lower panels presented the analyses of Lab-K$^+$ and ECG-K$^+$. The solid line and dashed line are point estimation and corresponding 95% conference interval, respectively. The baseline model of combined analysis is adjusted to each hospital site and based on Cox proportional hazard model or logistic regression as appropriate for each outcome.

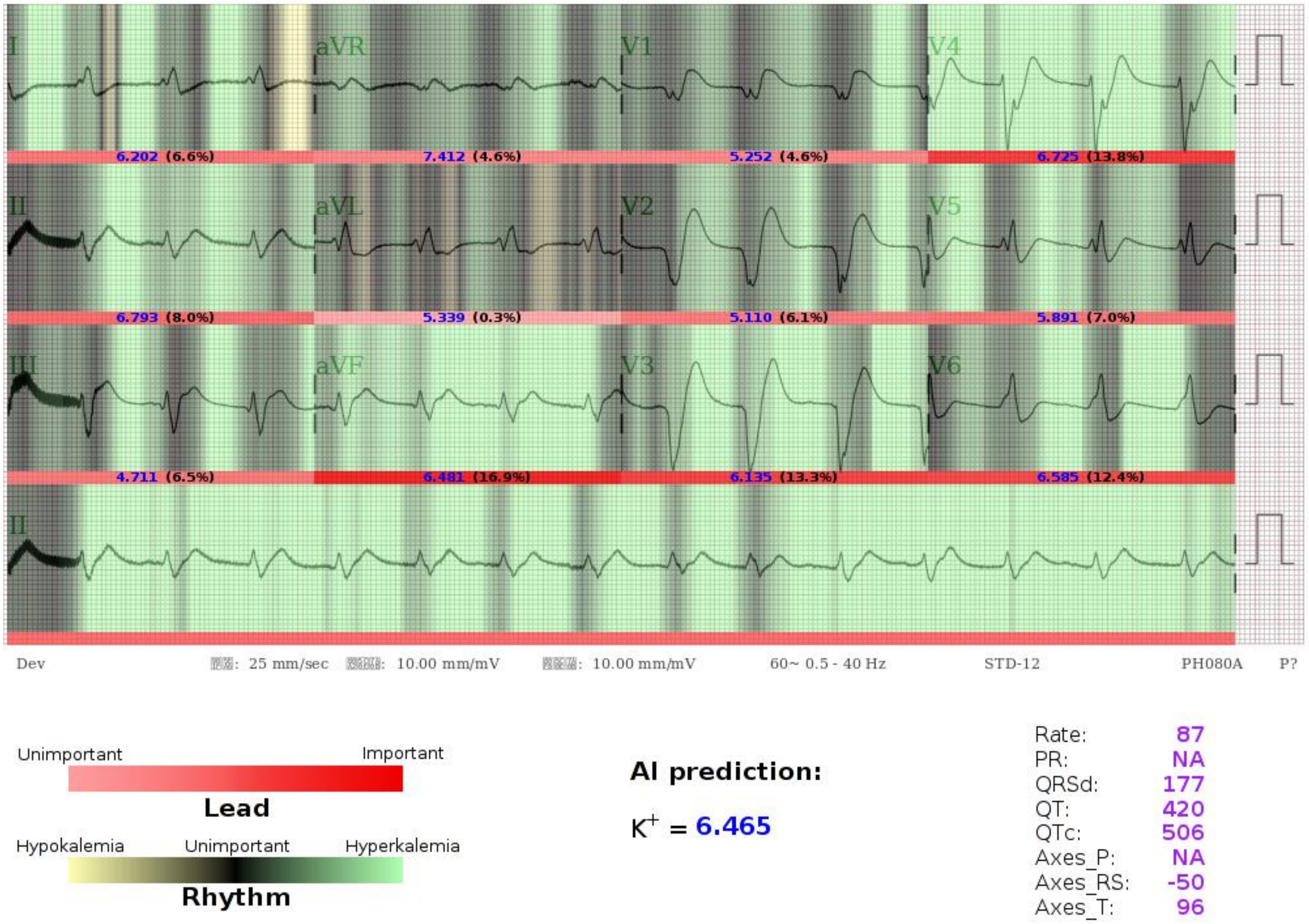

Supplementary Figure 10 | AI-ECG visualization of a patient with ECG-hyperkalemia and Lab-normokalemia (Case 1). A 34-year-old man was taken to our ED after attempted suicide with carbon monoxide poisoning. On physical examination, his blood pressure was 146/86 mmHg and he was comatose with GCS of E1M1Vt. ECG12Net predicted a very high value of ECG-K^+ concentration (6.465 mmol/L) based on the junctional rhythm, complete left bundle branch block, and prolonged corrected QT interval. The laboratory potassium was 4.5 mmol/L. The patient expired 8 days later due to hypoxic encephalopathy and multiple organ dysfunction.

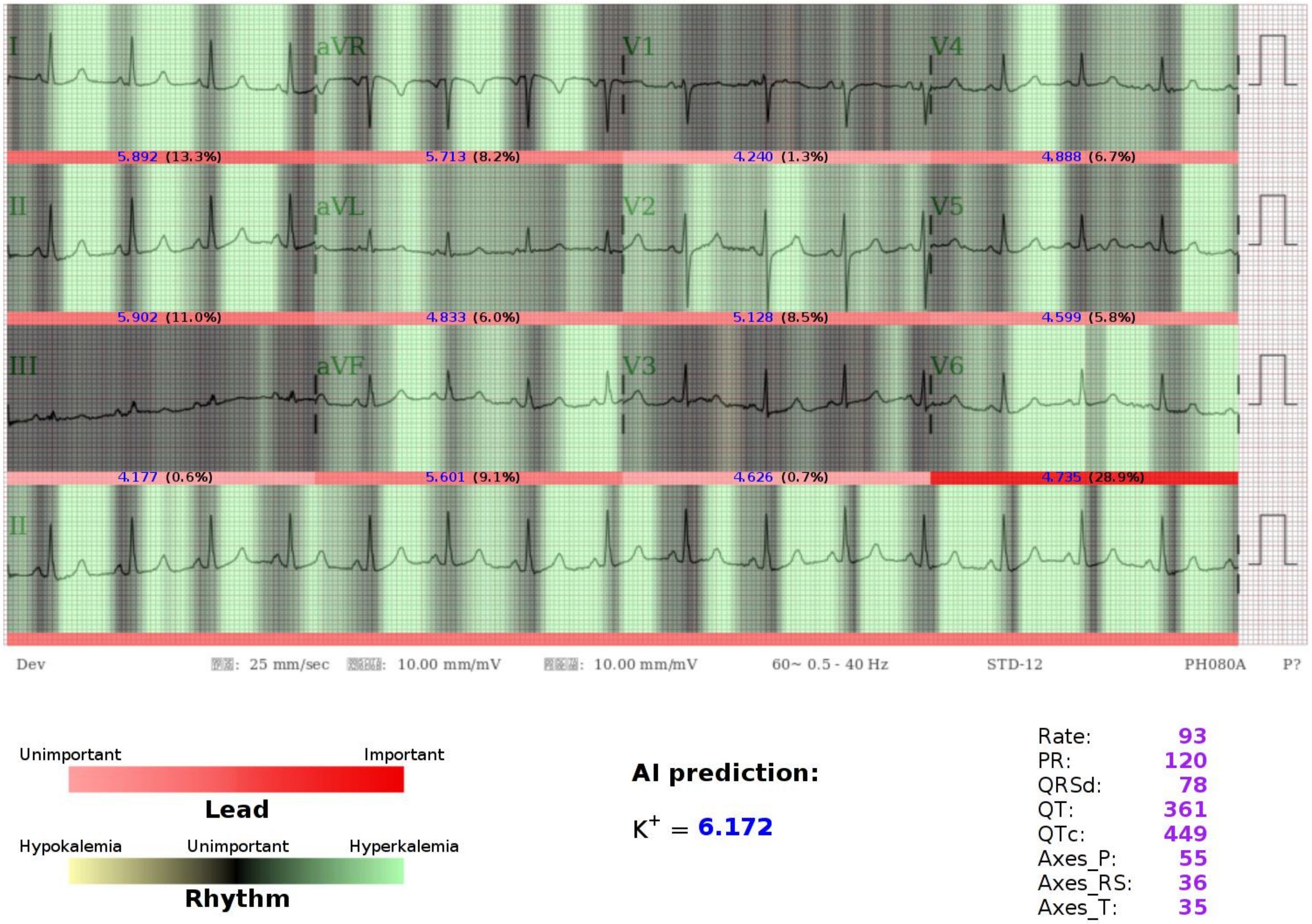

Supplementary Figure 11 | AI-ECG visualization of a patient with ECG-hyperkalemia and Lab-normokalemia (Case 2). A 50-year-old woman with advanced liver cirrhosis from alcohol use disorder and hepatitis C presented to the ED with hematemasis. On physical examination, her blood pressure was 120/94 mmHg with heart rate of 91 beats per minutes. Pale conjunctiva, icteric sclera and ovoid abdomen with shifting dullness were identified. Pertinent laboratory findings included hemoglobin 6.1 g/dL, platelet 81,000/mL, prothrombin time 17.8 seconds, total bilirubin 4.1 mg/dL, aspartate aminotransferase 109 U/L, alanine transaminase 28 U/L, and albumin 1.7 g/dL. The ECG revealed sinus tachycardia and non-specific ST-T wave changes. Accordingly, a high ECG-K^+ concentration (6.172 mmol/L) was predicted. The laboratory potassium was 5 mmol/L. The patient expired in the hospital 18 days later due to massive bleeding from an esophageal-gastric junction ulcer and aspiration pneumonia with respiratory failure. The patient's family requested no resuscitation due to her advanced stage of liver cirrhosis.

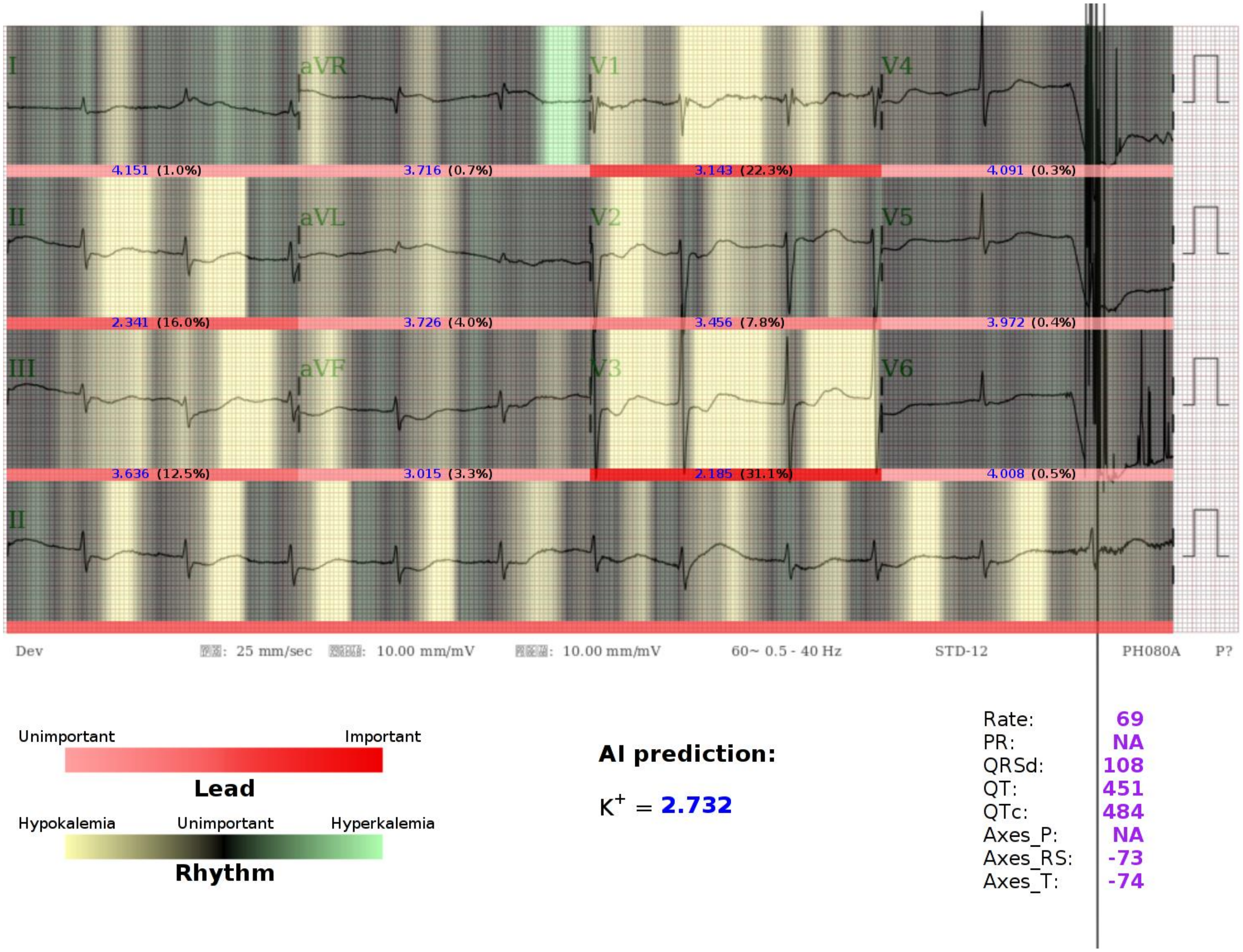

Supplementary Figure 12 | AI-ECG visualization of a patient with ECG-hypokalemia and Lab-normokalemia (Case 3). An 83-year-old man with a history of chronic obstructive pulmonary disease, valvular heart disease with severe mitral regurgitation, and atrial fibrillation presented to the ED with altered mental status and chest tightness for 2 days. On physical examination, irregular heart beats with grade III/VI pansystolic murmur over the left sternal border were noted. The ECG revealed atrial fibrillation with rate of 66 beats/min, ST depression in the precordial and limb leads, and prolonged corrected QT interval for rate. A low ECG-K^+ concentration (2.732 mmol/L) was predicted based on the ST depressions and prolonged QT intervals in V1-V3, and leads II, and III. The laboratory potassium was 3.9 mmol/L. The patient expired in the hospital 4 days later due to sudden cardiac arrest. The patient's family had declined resuscitation due to his multiple co-morbidities and advanced age.

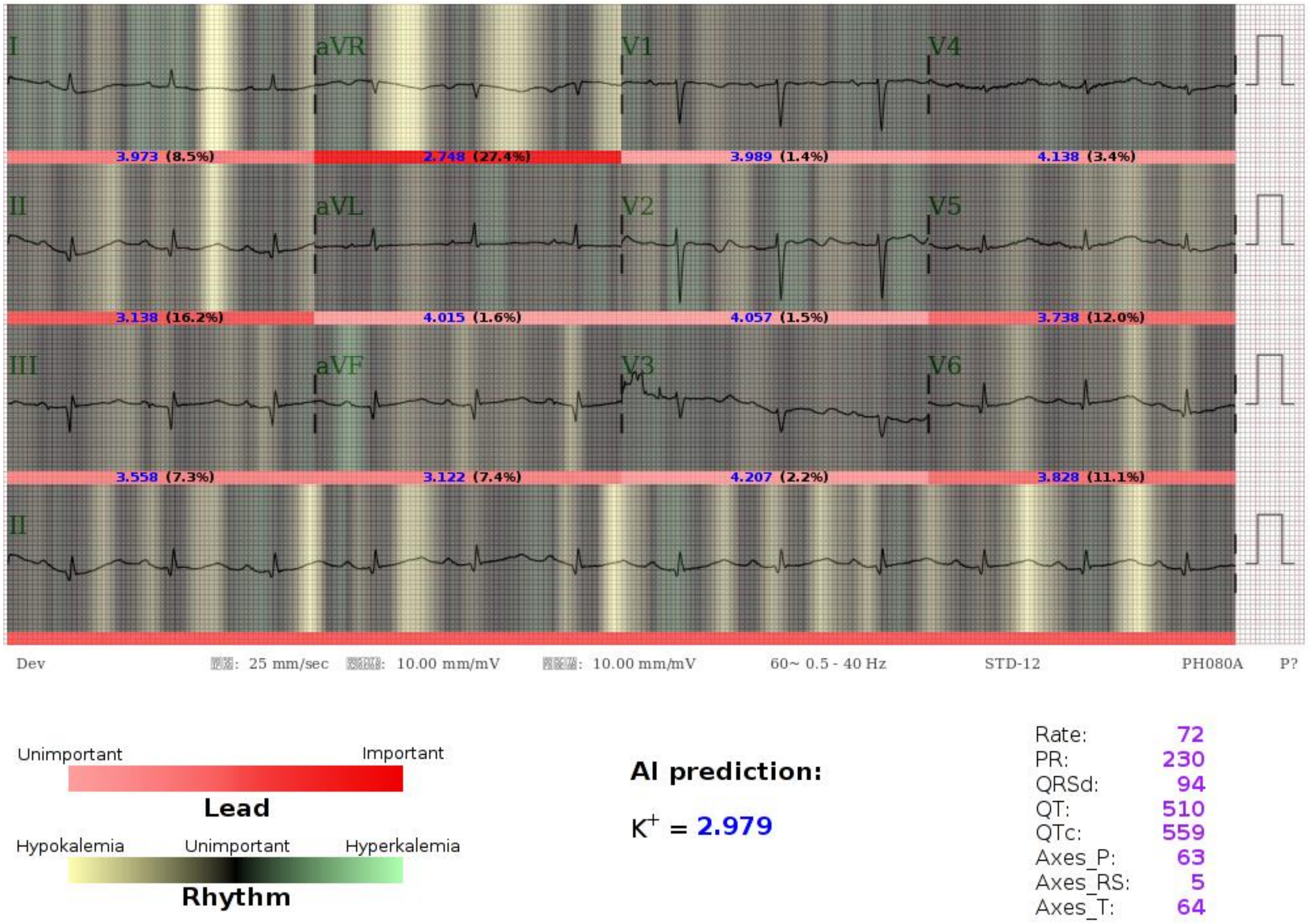

Supplementary Figure 13 | AI-ECG visualization of a patient with ECG-hypokalemia and Lab-normokalemia (Case 4). A 79-year-old man with a history of metastatic colon adenocarcinoma to the liver presented to the ED with generalized weakness and chills for one day. On physical examination, his blood pressure was 112/70 mmHg. His consciousness was alert with bilateral muscle power of 4/5. Ovoid abdomen with shifting dullness was noted. The ECG revealed sinus rhythm, poor R wave progression in the precordial leads, and prolonged corrected QT interval. Accordingly, a low value ECG-K^+ concentration (2.979 mmol/L) was predicted. The laboratory potassium was 3.9 mmol/L. The patient expired 19 days later due to sepsis and cancer progression.
