# Supplementary material for: Point-of-care artificial intelligence-enabled ECG for dyskalemia: a retrospective cohort analysis for accuracy and outcome prediction
Source: NPJ Digit Med. 2022 Jan 19;5:8. doi: 10.1038/s41746-021-00550-0 (PMC8770475; doi:10.1038/s41746-021-00550-0)
Supplement: Supplementary file 2 — Reporting Summary [file 41746_2021_550_MOESM2_ESM.pdf]

nature portfolio

Corresponding author(s): Shih-Hua Lin

Last updated by author(s): Dec 10, 2021

Reporting Summary

Nature Portfolio wishes to improve the reproducibility of the work that we publish. This form provides structure for consistency and transparency in reporting. For further information on Nature Portfolio policies, see our Editorial Policies and the Editorial Policy Checklist.

Statistics

For all statistical analyses, confirm that the following items are present in the figure legend, table legend, main text, or Methods section.

n/a	Confirmed	
☐	☒	The exact sample size (n) for each experimental group/condition, given as a discrete number and unit of measurement
☐	☒	A statement on whether measurements were taken from distinct samples or whether the same sample was measured repeatedly
☐	☒	The statistical test(s) used AND whether they are one- or two-sided<br>*Only common tests should be described solely by name; describe more complex techniques in the Methods section.*
☐	☒	A description of all covariates tested
☒	☐	A description of any assumptions or corrections, such as tests of normality and adjustment for multiple comparisons
☐	☒	A full description of the statistical parameters including central tendency (e.g. means) or other basic estimates (e.g. regression coefficient) AND variation (e.g. standard deviation) or associated estimates of uncertainty (e.g. confidence intervals)
☐	☒	For null hypothesis testing, the test statistic (e.g. F, t, r) with confidence intervals, effect sizes, degrees of freedom and P value noted<br>*Give P values as exact values whenever suitable.*
☒	☐	For Bayesian analysis, information on the choice of priors and Markov chain Monte Carlo settings
☒	☐	For hierarchical and complex designs, identification of the appropriate level for tests and full reporting of outcomes
☒	☐	Estimates of effect sizes (e.g. Cohen's d, Pearson's r), indicating how they were calculated

Our web collection on statistics for biologists contains articles on many of the points above.

Software and code

Policy information about availability of computer code

Data collection	All data utilized in this study were electronically retrieved. R, FTP, SQL and other core systems of our data warehouse were utilized.
Data analysis	Data analysis R version 3.4.4; Model training and inference R version 3.4.4 and Mxnet version 1.3.0.

For manuscripts utilizing custom algorithms or software that are central to the research but not yet described in published literature, software must be made available to editors and reviewers. We strongly encourage code deposition in a community repository (e.g. GitHub). See the Nature Portfolio guidelines for submitting code & software for further information.

Data

Policy information about availability of data

All manuscripts must include a data availability statement. This statement should provide the following information, where applicable:

- Accession codes, unique identifiers, or web links for publicly available datasets
- A description of any restrictions on data availability
- For clinical datasets or third party data, please ensure that the statement adheres to our policy

The data analyzed in this study is not publicly available due to privacy and security concerns. The data may be shared with a third party upon execution of data sharing agreement for reasonable requests, such requests should be addressed to C. Lin or S.H. Lin.

Field-specific reporting

Please select the one below that is the best fit for your research. If you are not sure, read the appropriate sections before making your selection.

☒ Life sciences ☐ Behavioural & social sciences ☐ Ecological, evolutionary & environmental sciences

For a reference copy of the document with all sections, see nature.com/documents/nr-reporting-summary-flat.pdf

Life sciences study design

All studies must disclose on these points even when the disclosure is negative.

Sample size

This study does not follow the traditional null hypothesis testing strategy that lends itself to power calculation (i.e., there is no null hypothesis for the algorithm). Instead, we abstracted all of the cases that met inclusion criteria and utilized measures of statistical precision. For sample sizes over 2,000 (>30,000 in this study), which is this case for this study, precision of proportions (i.e., AUC, accuracy, sensitivity, etc) have precision <1 percentage point. Thus, the sample size of the study was sufficiently sized to support the research objectives.

Data exclusions

There were 50,358 and 8,223 ED visits to the participating medical center and community hospital, respectively. All ED visits started with an initial assessment at the triage station. Those whose interval between ECG and Lab-K+ measurement was longer than 1 hour were also excluded, due to the possibility of having received treatments which alter the ECG during the long time interval. In total, 34,803 ED visits from 26,499 patients to the medical center and 6,492 visits from 4,747 patients to the community hospital were recruited for analysis.

Replication

The study design included a pre-trained model based on data before 2016, and the patients included in the independent test data were after May, 2019. Moreover, an external test set from a different hospital was also used to validate its replicaction.

Randomization

This was a retrospective chart review study. There was no intervention or treatment assignment as a result. Therefore, randomization is not applicable.

Blinding

As noted in the randomization section above, there was no randomization or treatment assignment. Accordingly, blinding was not applicable to this study.

Reporting for specific materials, systems and methods

We require information from authors about some types of materials, experimental systems and methods used in many studies. Here, indicate whether each material, system or method listed is relevant to your study. If you are not sure if a list item applies to your research, read the appropriate section before selecting a response.

Materials & experimental systems

n/a	Involved in the study	
☒	☐	Antibodies
☒	☐	Eukaryotic cell lines
☒	☐	Palaeontology and archaeology
☒	☐	Animals and other organisms
☐	☒	Human research participants
☒	☐	Clinical data
☒	☐	Dual use research of concern

Methods

n/a	Involved in the study	
☒	☐	ChIP-seq
☒	☐	Flow cytometry
☒	☐	MRI-based neuroimaging

Human research participants

Policy information about studies involving human research participants

Population characteristics

This study population consisted of emergency department patients seen at Tri-Service General Hospital health care system in Taipei, Taiwan. Eligible patients were required to have at least one digital, standard 10-second 12-lead ECG acquired in the supine position between May 2019 and December 2020 and at least one serum laboratory potassium measurement within 1 hours after triage station. The overall patient populations had mean age of 61.3+/-19.5 and 65.2+/-21.3 in an academic medical center (NeiHu General Hospital) and a community hospital (Tingzhou Branch Hospital), respectively, and ~20%/~2% of the population had hypo/hyperkalemia.

Recruitment

N/A - records based research

Ethics oversight

This study was approved by the institutional review board of Tri-Service General Hospital, Taipei, Taiwan (IRB NO. C202005055). Patients' consent was waived because data were collected retrospectively and in anonymized files and encrypted from the hospital to the data controller.

Note that full information on the approval of the study protocol must also be provided in the manuscript.
